# Diversity and evolution of phycobilisomes in marine *Synechococcus *spp.: a comparative genomics study

**DOI:** 10.1186/gb-2007-8-12-r259

**Published:** 2007-12-05

**Authors:** Christophe Six, Jean-Claude Thomas, Laurence Garczarek, Martin Ostrowski, Alexis Dufresne, Nicolas Blot, David J Scanlan, Frédéric Partensky

**Affiliations:** 1 UMR 7144 Université Paris VI and CNRS, Station Biologique, Groupe Plancton Océanique, F-29682 Roscoff cedex, France; 2 Mount Allison University, Photosynthetic Molecular Ecophysiology Group, Biology Department, E4L 1G7 Sackville, New Brunswick, Canada; 3 UMR 8186 CNRS and Ecole Normale Supérieure, Biologie Moléculaire des Organismes Photosynthétiques, F-75230 Paris, France; 4 Department of Biological Sciences, University of Warwick, Coventry CV4 7AL, UK

## Abstract

**Background:**

Marine *Synechococcus *owe their specific vivid color (ranging from blue-green to orange) to their large extrinsic antenna complexes called phycobilisomes, comprising a central allophycocyanin core and rods of variable phycobiliprotein composition. Three major pigment types can be defined depending on the major phycobiliprotein found in the rods (phycocyanin, phycoerythrin I or phycoerythrin II). Among strains containing both phycoerythrins I and II, four subtypes can be distinguished based on the ratio of the two chromophores bound to these phycobiliproteins. Genomes of eleven marine *Synechococcus *strains recently became available with one to four strains per pigment type or subtype, allowing an unprecedented comparative genomics study of genes involved in phycobilisome metabolism.

**Results:**

By carefully comparing the *Synechococcus *genomes, we have retrieved candidate genes potentially required for the synthesis of phycobiliproteins in each pigment type. This includes linker polypeptides, phycobilin lyases and a number of novel genes of uncharacterized function. Interestingly, strains belonging to a given pigment type have similar phycobilisome gene complements and organization, independent of the core genome phylogeny (as assessed using concatenated ribosomal proteins). While phylogenetic trees based on concatenated allophycocyanin protein sequences are congruent with the latter, those based on phycocyanin and phycoerythrin notably differ and match the *Synechococcus *pigment types.

**Conclusion:**

We conclude that the phycobilisome core has likely evolved together with the core genome, while rods must have evolved independently, possibly by lateral transfer of phycobilisome rod genes or gene clusters between *Synechococcus *strains, either via viruses or by natural transformation, allowing rapid adaptation to a variety of light niches.

## Abstract 2

By comparing Synechococcus genomes, candidate genes required for the production of phycobiliproteins, which are part of the light-harvesting antenna complexes called phycobilisomes, were identified. Phylogenetic analyses suggest that the phycobilisome core evolved together with the core genome, whereas rods evolved independently.

## Background

Since their discovery almost 30 years ago [1,2], marine representatives of the *Synechococcus *genus have been found in the upper illuminated layer of most marine ecosystems, from coastal to offshore waters as well as from low to high latitudes [3-5]. Besides being ubiquitous, *Synechococcus *are often abundant, with cell densities ranging from a few hundred to over one million cells per milliliter of seawater [6-10].

*Synechococcus *cells owe their vivid colors mainly to their photosynthetic antenna, called phycobilisomes (PBSs). These water-soluble macromolecular complexes comprise rods surrounding a central core and are made of phycobiliproteins, which covalently bind chromophores (phycobilins) by thioether bonds to cysteinyl residues (for reviews, see [11-15]). All phycobiliproteins in cyanobacteria consist of two distinct subunits, α and β, organized either as trimeric (αβ)_3 _or, in most cases, as hexameric discs (αβ)_6_. The PBS core of marine *Synechococcus *is made of allophycocyanin (AP), which binds only the blue-colored chromophore phycocyanobilin (PCB; *A*_max _= 620 nm). In some strains, phycocyanin (PC) may constitute the whole rod, as it does in many freshwater cyanobacteria (for example, *Synechococcus elongatus *PCC 7942, *Synechocystis *sp. PCC 6803). In that case, it binds only PCB and is of the C-PC type [15]. However, in most phycoerythrin (PE)-containing marine *Synechococcus *characterized so far, PC makes up the basal disc at the core-proximal end of the rods. It binds both PCB and the red-colored chromophore phycoerythrobilin (PEB; *A*_max _= 550 nm) at a molar ratio of 1:2 and thus belongs to the R-PCII type [16]. In strain WH7805, however, the base of the rods is thought to consist of a so-called R-PCIII, an optically unusual PC that binds PCB and PEB at a molar ratio of 2:1 [15,17].

In most PE-containing *Synechococcus *strains isolated to date, the distal part of the PBS rods is composed of two types of PE (PEI and PEII). PEII always binds both PEB and the orange colored phycourobilin (PUB; *A*_max _= 495 nm), whereas PEI binds either only PEB or both PEB and PUB [18,19]. However, Everroad and Wood [20] have recently suggested that some marine *Synechococcus *strains may contain rods with a single type of PE that binds only PEB chromophores. In addition, the higher order structure of PBSs is stabilized by linker polypeptides that contribute to the building of a protein environment around the phycobilins [14,21]. These linkers have very variable sizes (8-120 kDa) but most are in the 27-35 kDa range. In the rods, only those associated with PEII are chromophorylated with PUB [19,21].

Although the *Synechococcus *genus itself is polyphyletic, marine *Synechococcus *characterized thus far form a well-defined branch within the cyanobacteria radiation, together with the *Prochlorococcus *and *Cyanobium *genera [22-25]. This grouping, called 'Cluster 5' by Herdman and coworkers [26], is a combination of the former Marine Clusters A and B previously defined by Waterbury and Rippka [27]. Cluster 5 thus gathers coastal, euryhaline *Synechococcus *strains as well as strictly marine strains (that is, with elevated growth requirements for Na^+^, Mg^+ ^and Ca^++^). Subclusters 5.1 and 5.2 have also been tentatively defined by Herdman and coworkers [26] in order to separate the strictly marine PE-containing strains (5.1) from a group of euryhaline strains lacking PE (5.2), including WH5701 and WH8007. However, Fuller and coworkers [23] have shown that one clade within the subcluster 5.1 (clade VIII) gathers euryhaline strains lacking PE and Chen and coworkers [25] have isolated several new members of subcluster 5.2 into culture that do contain PE. Furthermore, the latter authors suggested that WH5701 and WH8007 might actually belong to two distinct clusters.

Among the strains containing two PE types, there is a clear consistency between phylogenies based on different molecular markers, including *rpoC1 *[28], *ntcA *[29], the 16S rRNA gene [23] and the 16S-23S rDNA internal transcribed spacer [24]. However, none of these phylogenies is congruent with the whole cell ratio of PUB to PEB. This chromophore ratio is known to vary according to the light niche, with open ocean strains predominantly displaying a high PUB:PEB whereas mesotrophic or coastal strains generally have lower ratios or no PUB [6,7,30-32]. Some strains even display a variable PUB:PEB depending on the ambient light quality, that is, they are able to chromatically adapt [33]. These so-called type IV chromatic adapters are not confined to a particular phylogenetic clade within Cluster 5 [34]. This raises the question of the molecular basis of differences in whole cell PUB:PEB between *Synechococcus *strains. More generally, one might wonder whether PBS components have undertaken a different evolutionary trajectory compared to the core genome.

In order to address these questions, we studied 11 *Synechococcus *strains, belonging to various phylogenetic clades according to Fuller *et al*. [23] and representing the whole variety of PBS pigmentations known so far within Cluster 5. We compared the PBS gene complements of these strains, an approach that revealed a number of novel PBS genes, including putative lyases and linker polypeptides. By combining these genomic data with biochemical and optical properties of isolated phycobiliprotein complexes, we identified several marine *Synechococcus *pigment types and we propose putative, structural models for their corresponding PBSs. We also examined the phylogeny of each phycobiliprotein type, yielding new insights into the evolution of PBS complexes within the marine *Synechococcus *group.

## Results

### *Synechococcus *pigment types

**Figure 1:**
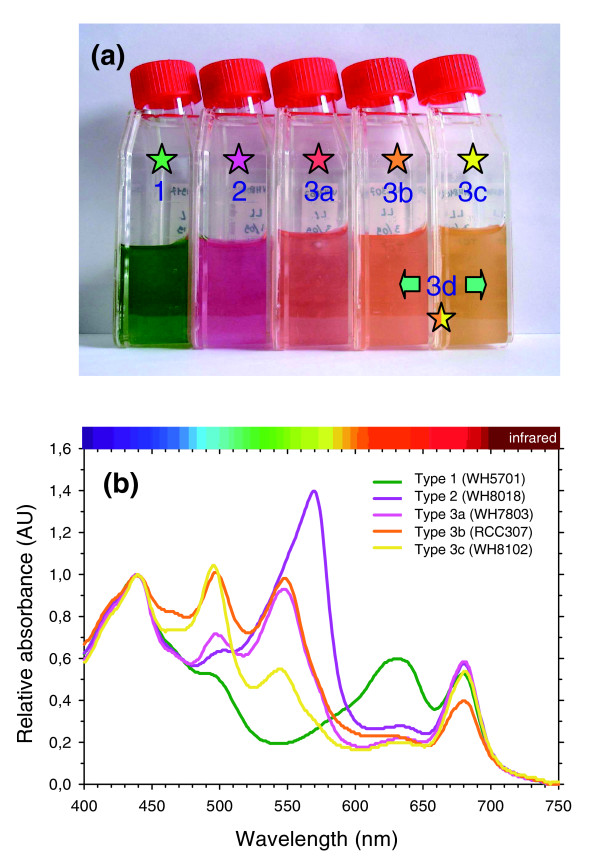
The diversity of pigment types among marine *Synechococcus *spp. **(a) **Photograph of representative cultured strains of the major pigment types (1-3) and subtypes (3a-c) of marine *Synechococcus *grown under low white light and **(b) **corresponding absorption properties of whole cells. Pigment subtype 3d corresponds to type IV chromatic adapters, which are able to modify their PBS pigmentation from subtype 3b when grown under white or green light to subtype 3c when grown under blue light. The different colors of stars in panel A are a code for the different pigment types.

Despite the apparently large diversity of pigmentation existing among marine *Synechococcus*, these can be partitioned into only three major types based on the phycobiliprotein composition of the rods: type 1 representatives have only PC, type 2 have PC and PEI and type 3 have PC, PEI and PEII. Type 3 can be further subdivided into four subtypes (3a through 3d) based on the ratio of the two chromophores (PEB and PUB) bound to PEs, a ratio that can be low, medium, high or variable. Figure 1a illustrates these different pigment types or subtypes and their corresponding colors. The 11 fully sequenced marine *Synechococcus *strains cover the whole range of PBS pigmentation known so far in this group [6,23,33]. Pigment type 1 is represented by the blue-green, PE-lacking strains WH5701 and RS9917. These strains absorb light optimally in the wavelength range 600-660 nm, that is, red and orange light (Figure 1b). The genome of the fuchsia pink WH7805 strain (pigment type 2) contains a single set of PE genes encoding a PEI-like complex, as detailed below. The whole cell absorption maximum of this form of PE devoid of PUB (*A*_max _= 570 nm, corresponding to yellow-green light) is red-shifted relative to other PEs (Figure 1b).

All strains displaying pigment type 3 possess both PEB and PUB chromophores. Subtypes 3a through 3c differ from one another in their whole cell ratio of PUB to PEB (hereafter PUB:PEB), as assessed by their fluorescence excitation maxima (*F*_495 nm _: *F*_550 nm_) with emission at 580 nm (Table 1). Note that the use of this fluorescence excitation ratio is preferable to using the corresponding absorption ratio (*A*_495 nm_: *A*_550 nm_) to characterize these different subtypes *in vivo*, since the carotenoids zeaxanthin and β-carotene have a notable contribution to the wavelength range of the PUB absorption peak (Figure 1b). The PUB:PEB can be either low (approximately 0.4) in type 3a strains such as WH7803, medium (approximately 0.8) in type 3b strains such as RCC307 or high (>1.7) in type 3c strains such as in WH8102 and CC9605 (Table 1). Depending on this ratio, PBSs of these strains preferentially harvest either green light (550 nm) or blue-green light (495 nm) (Figure 1b). Finally, pigment type 3d includes strains with a variable PUB:PEB (0.7-1.7), depending on whether these cells are grown under white/green or blue light [33,34]. These type IV chromatic adapters include the strains CC9311, RS9916, BL107 and CC9902 as well as a number of other strains that have not yet been sequenced (including WH8020, M16.17, M11.1, RCC61 (a.k.a. Minos 11) and RS9911; Table 1 and data not shown). To this suite of pigment types can be added a 'moderately high' PUB:PEB subtype (PUB:PEB approximately 1.2), represented by strain WH8103 and which is indistinguishable by eye from, and included within, type 3c (Figure 1a). Though as yet unsequenced, the genome of WH8103 has been screened, in part, by suppression subtractive hybridization [35].

**Table 1:** Strain numbers, phylogenetic position and PBS characteristics of all marine *Synechococcus *spp. mentioned in this paper

Strain name	RCC number	Subcluster	Clade	Pigment type	PUB:PEB	PEI form	PEII form	PC form	References
WH5701^†^	1,084	5.2	NA	1	NA	NA	NA	C-PC	PC: this paper
RS9917^†^	556	5.1	VIII	1	NA	NA	NA	C-PC	PC: this paper
WH7805^†^	1085	5.1	VI	2	NA	A*	NA	R-PCIII	PC: [17] PE: [36]
WH8018	649	5.1	VI	2	NA	A*	NA	C-PC	PC: this paper PE: [36]
WH7803^†^	752	5.1	V	3a	WL: 0.440 ± 0.004 BL: 0.443 ± 0.006	A	A	R-PCII	PC: [16] PE: [18]
Almo03	43	5.1	I	3a	WL: 0.417 ± 0.017 BL: ND	A	A	ND	PE: this paper
RS9912	551	5.1	II	3a	WL: 0.435 ± 0.004 BL: 0.438 ± 0.003	A	A	ND	PE: this paper
RCC307^†^	307	5.1	X	3b	WL: 0.775 ± 0.103 BL: 0.761 ± 0.002	WL: A BL: ND	WL: B BL: ND	ND	PE: this paper
CC9311^†^	1,086	5.1	I	3d (CA)	WL: 0.719 ± 0.060 BL: 1.603 ± 0.023	ND	ND	ND	-
CC9902^†^	-	5.1	IV	3d (CA)	Variable between WL and BL	ND	ND	ND	B Palenik, personal communication
BL107^†^	515	5.1	IV	3d (CA)	WL: 0.735 ± 0.003 BL = 1.695 ± 0.149	ND	ND	ND	-
RS9916^†^	555	5.1	IX	3d (CA)	WL: 0.733 ± 0.003 BL: 1.659 ± 0.054	ND	WL: B BL:ND	ND	PE: this paper
WH8020	751	5.1	I	3d (CA)	WL: 0.737 ± 0.003 BL:1.626 ± 0.042	WL: A BL: ND	WL: B BL: ND	R-PCII	PC: [16] PE: [18]
M11.1	790	5.1	-	3d (CA)	WL: 0.731 ± 0.004 BL: 1.849 ± 0.101	WL: B BL: B	WL: B BL: C	ND	PE: [34]
M16.17	793	5.1	-	3d (CA)	WL: 0.719 ± 0.015 BL: 1.826 ± 0.140	WL: B BL: B	WL: B BL: C	ND	PE: [34]
WH8103	29	5.1	III	3c	WL: 1.156 ± 0.014 BL: 1.154 ± 0.012	B	C	R-PCII	PC: [16] PE: [18]
WH8102^†^	539	5.1	III	3c	WL: 1.856 ± 0.117 BL: 1.903 ± 0.128	B	C	ND	PE: [19]
CC9605^†^	753	5.1	II	3c	WL: 2.136 ± 0.083 BL: 1.999 ± 0.187	B	C	ND	PE: this paper
Oli31	44	5.1	VII	3c	WL: 1.741 ± 0.012 BL: 1.774 ± 0.046	B	C	ND	PE: this paper

Subcluster and clade numbers are as defined in [23]. Strains are ordered by pigment type (1-3), as defined by their PBS rod composition, and subtype (3a-d) as defined by their whole cell PUB to PEB fluorescence excitation ratio (PUB:PEB ± standard deviation; *n *= 2 to 4). Phycobiliproteins have been classified into different forms, based on their respective chromophorylation (see text). References in the last column specify which PBP is described in which publication. CA, type IV chromatic adapter; A*, red-shifted PE; NA, not applicable; ND, not determined; WL, white light acclimation; BL, blue light acclimation. ^†^Sequenced genomes.

### Optical properties of phycobiliproteins

**Figure 2:**
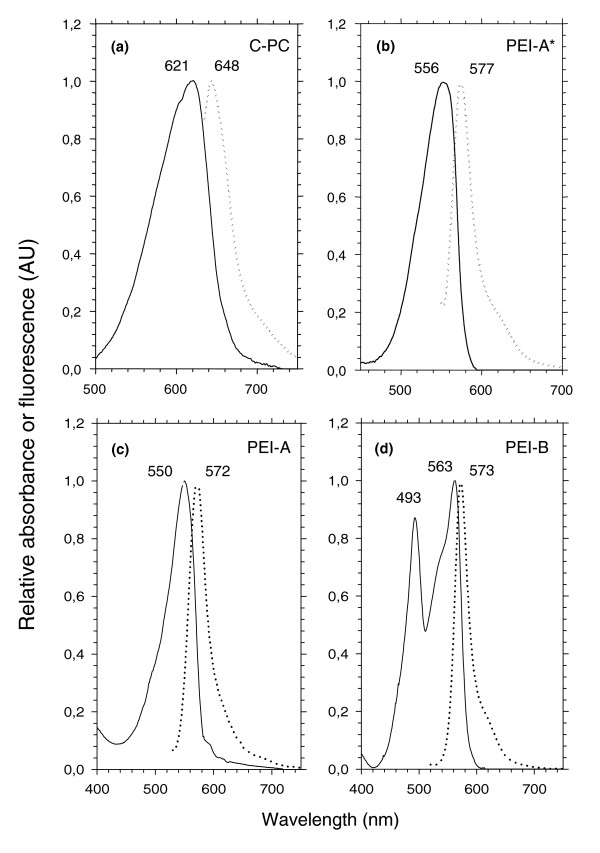
Absorption (continuous line) and fluorescence (dotted line) properties of isolated PBP complexes. **(a) **C-PC (as in *Synechococcus *spp. RS9917, WH5701 and WH8018); **(b) **PEI-A* (as in *Synechococcus *spp. WH8018 and WH7805); **(c) **PEI-A (as in *Synechococcus *spp. WH7803, Almo03 and RS9912); **(d) **PEI-B (as in *Synechococcus *spp. WH8102, CC9605 and Oli31).

**Figure 3:**
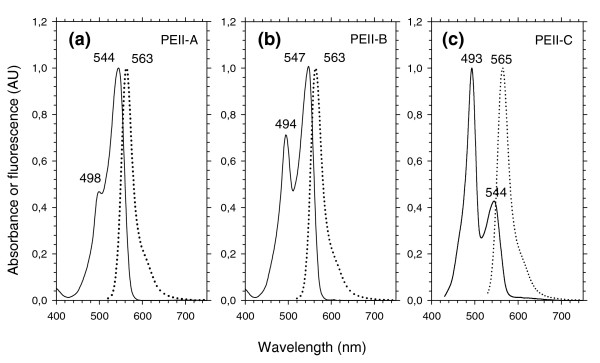
Absorption (continuous line) and fluorescence (dotted line) properties of the isolated PEII complexes. **(a) **PEII-A (as in *Synechococcus *sp. WH7803); **(b) **PEII-B (as in *Synechococcus *sp. RCC307); **(c) **PEII-C (as in *Synechococcus *spp. CC9605 and WH8102). Type IV chromatic adapters have a PEII-B under white or green light and a PEII-C under blue light [34].

The color and specific absorption properties of whole *Synechococcus *cells (Figure 1) are mainly determined by the major phycobiliprotein form occurring in the PBS rods. Isolated PC and/or PE complexes have been previously characterized in a few marine *Synechococcus *strains, including WH7803, WH7805, WH8102, WH8103 and the chromatic adapters WH8020 (under white light only), M11.1 and M16.17 [13,16-19,34,36], as summarized in Table 1. In order to explore further the diversity and possible combinations of these phycobiliproteins in the different *Synechococcus *pigment types, we have used sucrose density gradients and isoelectric focusing to isolate PC, PEI and/or PEII from a number of other strains and then have determined their optical properties (Figures 2 and 3 and Table 1).

The PC present in WH5701 and RS9917, which formed a sky blue band on isoelectric focusing gels (not shown), had absorption (*A*_max _= 621 nm) and fluorescence (*F*_max _= 648 nm) properties typical of C-PC (Figure 2a), that is, known to bind only PCB chromophores [15]. We also found C-PC in the PE-containing, PUB-lacking strain WH8018, whereas WH7805 (which, like WH8018, displays pigment type 2) is known to possess R-PCIII [17]. R-PCIII has a molar PCB:PEB of 2:1, like the R-PCI of red algae, but a different spectrum, with an *A*_max _at 555 nm and a shoulder at 590 nm [17]. Our isolation protocol did not allow us to obtain a pure PC fraction from any of the PEII-containing strains, because the PC band was always contaminated by variable amounts of PEII. It is known, however, that *Synechococcus *sp. WH7803, like WH8020 and WH8103, possesses a R-PCII type PC with a molar PEB:PCB of 2:1; it has absorption peaks at 533, 554 and 615 nm and maximal fluorescence emission at 646 nm [16].

Several types of PEI can be distinguished based on their different optical properties. The major phycobiliprotein found in WH7805 and WH8018, a PEI-like phycobiliprotein, exhibited an *A*_max _at 556 nm and an *F*_max _at 577 nm (Figure 2b). We have called it PEI-A* to distinguish it from the PEI-A found in *Synechococcus *strains displaying the 3a and 3b pigment types. PEI-A has blue-shifted optical properties (*A*_max _= 550 nm; *F*_max _= 572 nm; Figure 2c) compared to PEI-A*, though both forms bind only PEB chromophores. PEI-B, which has a molar PUB:PEB of 2:3 [18], has been found in all strains exhibiting pigment type 3c examined thus far, as well as in some chromatic adapters, including M11.1 and M16.17 [34]. It has maximal absorption at 493 and 563 nm and fluorescence at 573 nm (Figure 2d).

Similarly, one can distinguish three optical types of PEII differing by their PUB:PEB. All have two absorption maxima (or at least shoulders) around 495 nm and 550 nm, due to the two chromophores they bind, and a maximal fluorescence emission around 565 nm. PEII-A (Figure 3a) is found only in *Synechococcus *pigment type 3a, including WH7803 [18], Almo03 and RS9912 (this study). Its molar PUB:PEB is most likely 1:5, although the cysteinyl site to which the sole PUB chromophore is bound (either α-75 or β-50/61) has not yet been ascertained. PEII-B (Figure 3b) is found in RCC307 (Table 1) and in all white light-grown chromatic adapters that have been screened thus far, including WH8020 [18], M11.1, M16.17 [34] and RS9916 (this study). Its molar PUB:PEB is 2:4. PEII-C (Figure 3c) is found in *Synechococcus *pigment type 3c, including WH8103 [18], WH8102 [19], Oli31 and CC9605 (this study) as well as in the blue light-grown chromatic adapters [34]. The molar PUB:PEB of this PEII has been shown to be 4:2 [18].

### Comparative analysis of the phycobilisome gene regions

**Figure 4:**
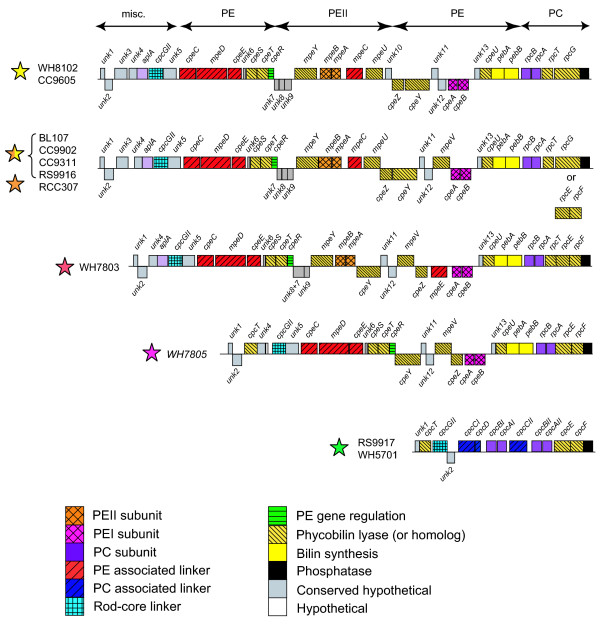
Comparison of PBS rod gene regions of the different pigment types of marine *Synechococcus*. Rectangles above and below the lines have a length proportional to the size of ORFs and correspond to the forward and the reverse strand, respectively. In several genomes, the sense of the rod region was inversed so that the regions all appear in the same direction. For the group formed by the chromatic adapters and RCC307, a few variations can be found with regard to the region shown here, which corresponds to BL107. First, the lyase-encoding gene(s) located near the 3'-end can either be a *rpcE-F *operon or *rpcG*, a *pecEF*-like fusion gene (see text). Second, the gene organization at the 5'-end can vary: *unk1 *is located elsewhere in the genome of RCC307 and the gene following *unk2 *is either the lyase gene *cpcT *in RS9916 and RCC307, *unk3 *in BL107 and CC9902, or none of these in CC9311. Colored stars indicate the pigment type of each strain (see Figure 1 for color code).

After careful annotation, we compared PBS gene complement (Additional data file 1) and organization in the 11 different genomes. One remarkable trait of marine *Synechococcus *is that most of the PBS genes are gathered into a few gene clusters [19,37]. As in several other cyanobacteria, a first small cluster groups together four AP core genes, in the order *apcE-A-B-C*, while two other core genes, *apcD *and *apcF *(encoding the minor α-B and β-18 AP subunits, respectively) have no PBS gene in their close vicinity. Most of the PBS rod genes are located in a much larger cluster, the size of which increases with the complexity of the rod structure from approximately 9-10 Kbp in pigment type 1 up to 27-28.5 Kbp in chromatic adapters (Figure 4). Interestingly, the gene organization in this region is very similar for strains belonging to a given pigment type. It is also similar between the chromatic adapters and the medium PUB:PEB strain RCC307.

In most genomes, the 5'-end of the PBS rod gene region starts with a short gene of unknown function (*unk1*). In RCC307, however, the *unk1 *ortholog is found elsewhere in the genome. The 3'-end of the region consists of a well conserved gene predicted to encode a low molecular weight phosphotyrosine phosphatase. In the blue-green, PE-lacking strains, the rest of the region is mainly composed of two identical *cpcB-A *operons encoding the C-PC α- and β-subunits and of genes encoding three rod linkers, one rod-core linker and two types of phycobilin lyases (CpcT and CpcE/F; see below). Both RS9917 and WH5701 have an additional *cpcB *gene copy outside the PBS rod gene region but, surprisingly, no additional *cpcA*.

A part of the PC gene cluster found in the blue-green strains (*cpcCI-D-B-A-CII*) is replaced in the fuchsia pink strain WH7805 by a set of 19 genes, likely involved in the synthesis and regulation of a PEI-like complex (Figure 2). The *pebA *and *pebB *genes, located at the 3'-end of this insertion, are known to be involved in the synthesis of PEB chromophores [38].

This PE region can also be found in all PEII-containing strains, but it is interrupted by an additional sub-region containing 5 to 9 genes, between the PE regulator *cpeR *[39] and the putative lyase gene *cpeY *in WH7803 (or *cpeZ *in the other strains). This inserted sub-region includes genes encoding the PEII α- and β-subunits, two phycobilin lyases, one linker polypeptide and two or three uncharacterized proteins. In addition, all PEII-containing strains have, upstream of *cpcGII*, an ortholog of *aplA*. Its product, AplA, which shows homology to the AP α-subunit (ApcA), was recently described in *Fremyella diplosiphon *as belonging to a new class of cyanobacterial photosensors of unknown function [40].

In the following sections, we have analyzed more specifically the phyletic profile (that is, the different patterns of occurrence of orthologs in the set of *Synechococcus *genomes) and characteristics of three gene categories: genes encoding linker polypeptides (Table 2), genes encoding putative phycobilin lyases (Table 3) and genes of unknown function specifically located in the PBS rod gene region and, therefore, potentially involved in PBS metabolism or regulation (Table 4).

**Table 2:** Presence or absence of genes encoding linker polypeptides in the different marine *Synechococcus *genomes

		Allophycocyanin		Phycocyanin			Phycoerythrin I		Phycoerythrin II				
					
Strain	Pigment type	*apcC *(L_C_)	*apcE *(L_CM_)	*cpcC *(L_R_)	*cpcD *(L_R_)	*cpcG *(L_RC_)	*cpeC *(L_R_)	*cpeE *(L_R_)	mpeD* (L_R_)^†^	*mpeC *(L_R_)^†^	*mpeE *(L_R_)^†^	*mpeF *(L_R_)^†^	*mpeG *(L_R_)^†^
WH5701	1	CC	CC^‡^	RC (*cpcCI*^‡^) RC (*cpcCII*^‡^) NC (*cpcCIII*^‡§^)	RC	GC (*cpcGI*)^‡ ^RC (*cpcGII*)	-	-	-	-	-	-	-
RS9917	1	CC	CC	RC (*cpcCI*) RC (*cpcCII*)	RC	GC (*cpcGI*) RC (*cpcGII*)	-	-	-	-	-	-	-
WH7805	2	CC	CC^‡^	-	-	GC (*cpcGI*)^‡ ^RC (*cpcGII*)	RC^‡^	RC^‡^	RC^‡^	-	-	-	-
WH7803	3a	CC	CC^‡^	-	-	GC (*cpcGI*)^‡ ^RC (*cpcGII*)	RC^‡^	RC^‡^	RC^‡^	-	RC^‡^	-	-
RCC307	3b	CC	CC^‡^	-	-	GC (*cpcGI*)^‡ ^RC (*cpcGII*)	RC^‡^	RC^‡^	RC^‡^	RC^‡^	NC^‡^	-	NC
CC9311	3d (CA)	CC	CC	-	-	GC (*cpcGI*) RC (*cpcGII*) NC (*cpcGIII*)	RC	RC	RC	RC	RC	NC	-
CC9902	3d (CA)	CC	CC	-	-	GC (*cpcGI*) RC (*cpcGII*) NC (*cpcGIII*)	RC	RC	RC	RC	NC	NC	-
BL107	3d (CA)	CC	CC	-	-	GC (*cpcGI*) RC (*cpcGII*) NC (*cpcGIII*)	RC	RC	RC	RC	NC	NC	-
RS9916	3d (CA)	CC	CC^‡^	-	-	GC (*cpcGI*)^‡ ^RC (*cpcGII*)	RC^‡¶^	RC^‡^	RC^‡^	RC^‡^	RC^‡^	-	NC
CC9605	3c	CC	CC	-	-	GC (*cpcGI*) RC (*cpcGII*) NC (*cpcGIII*)	RC	RC	RC	RC	NC	-	-
WH8102	3c	CC	CC	-	-	GC (*cpcGI*) RC (*cpcGII*)	RC	RC	RC	RC	NC	-	-

**Figure 5:**
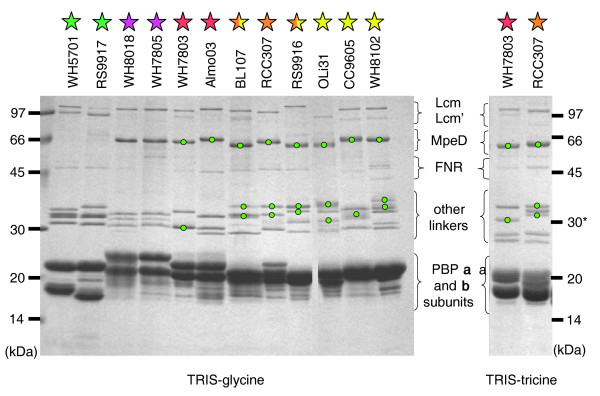
Coomassie blue stained LiDS polyacrylamide gradient (10-20%) gel of PBS linkers run using a Tris-glycine buffer system (left). A Tris-tricine buffer (right) gave higher band resolution for RCC307 and WH7803. Green dots indicate linker polypeptides fluorescing green under UV light due to the presence of a PUB chromophore. Colored stars indicate the pigment type of each strain (see Figure 1 for color code). FNR: ferredoxin:NADP^+ ^oxidoreductase.

CC, gene located within the PBS core gene cluster; GC, gene located within a cluster comprising the *cpcGI *and *cpcS *genes; NC, gene unlinked to other PBS genes; RC, gene located within the PBS rod gene cluster. Novel gene names proposed in this study are underlined. The linker polypeptide compositions of *Synechococcus *spp. WH5701, WH7803, WH7805 and RS9916 were checked by mass spectrometry after cutting the bands out of the LiDS-PAGE gel shown in Figure 5. For annotating paralogs that originated from recent gene duplications and have no obvious differential functional specializations (one-function paralog family), we chose the genetic nomenclature used by Berlyn [77] for *Escherichia coli *K-12. *MpeD is a chimeric protein made of two linker domains associated with PEI and PEII, respectively [19]. ^†^Linkers chromophorylated with PUB. ^‡^Linkers that have been identified by mass spectrometry. ^§^The product of this *cpcC *gene has an extended carboxyl terminus showing strong homology to CpcD. ^¶^CpeC and MpeC co-eluted in RS9916, explaining the darker band observed at approximately 36 kDa apparent molecular weight in Figure 5. CA, type IV chromatic adapter; L_C_, core linker; L_CM_, core-membrane linker; L_R_, rod linker; L_RC_, rod-core linker.

**Table 3:** Presence or absence of genes encoding putative phycobilin lyases in the different *Synechococcus *genomes

		Phycocyanin						Phycoerythrin I and/or II								
			
Strain	Pigment type	*cpcEF *operon	*rpcEF *operon	rpcG*	*cpcS*^†^	*cpcT*^‡^	*rpcT*^§^	*cpeS*	*cpeT*	*cpeU*^¶^	*cpeY*	*cpeZ*	*mpeV*	*mpeU*	*mpeY*^¥^	*mpeZ*^¥^
WH5701	1	RC	-	-	GC^#^	RC	-	-	-	-	-	-	-	-	-	-
RS9917	1	RC	-	-	GC	RC	-	-	-	-	-	-	-	-	-	-
WH7805	2	-	RC	-	GC	RC	-	RC	RC	RC	RC	RC	RC	-	-	-
WH7803	3a	-	RC	-	GC	-	RC	RC	RC	RC	RC	RC	RC	-	RC	-
RCC307	3b	-	RC	-	GC	RC	-	RC	RC	RC	RC	RC	RC	RC	RC	NC
CC9902	3d (CA)	-	RC	-	GC	-	RC	RC	RC	RC	RC	RC	RC	RC	RC	NC
CC9311	3d (CA)	-	RC	-	GC	-	RC	RC	RC	RC	RC	RC	RC	RC	RC	NC
BL107	3d (CA)	-	-	RC	GC	-	RC	RC	RC	RC	RC	RC	RC	RC	RC	NC
RS9916	3d (CA)	-	-	RC	GC	RC	RC	RC	RC	RC	RC	RC	RC	RC	RC	NC
CC9605	3c	-	-	RC	GC	-	RC	RC	RC	RC	RC	RC	-	RC	RC	-
WH8102	3c	-	-	RC	GC	-	RC	RC	RC	RC	RC	RC	-	RC	RC	-

GC, gene located within a cluster comprising the *cpcGI *and *cpcS *genes; NC, gene unlinked to other PBS genes; RC, gene located within the PBS rod gene cluster. Novel gene names proposed in this study are underlined. *pecE/F-like fusion gene [19]. ^†^Ortholog of *Nostoc *sp. PCC 7120 '*cpeS1*' gene [51,52] that we propose to rename *cpcS *(see text). ^‡^Ortholog of a *Synechococcus *sp. PCC 7002 *cpcT *gene [50]. ^§^Novel *cpcT *paralog found downstream of *rpcA*. ^¶^Novel *cpcS *paralog found upstream of *pebA*. ^¥^These two novel, closely related genes are both paralogs of *cpeY*. ^#^Gene split into two different reading frames in this strain. CA, type IV chromatic adapter.

**Table 4:** Presence or absence of genes encoding conserved hypothetical genes located in the phycobilisome rod gene region

Strain	Pigment type	*unk1*	*unk2*	*unk3*	*unk4*	*unk5*	*unk6*	*unk7*	*unk8*	*unk9*	*unk10*	*unk11*	*unk12*	*unk13*
WH5701	1	RC	RC	-	-	-	-	-	-	-	-	-	-	-
RS9917	1	RC	RC	-	-	-	-	-	-	-	-	-	-	-
WH7805	2	RC	RC	NC	RC	RC	RC	-	-	-	-	RC	RC	RC
WH7803	3a	RC	RC	NC	RC	RC	RC	RC (fused/inversed)		RC	-	RC	RC	RC
RCC307	3b	NC	RC	-	RC	RC	-	RC	RC	RC	NC	RC	RC	RC
CC9311	3d (CA)	RC	RC	NC	RC	RC	RC	RC	RC	RC	NC	RC	RC	RC
CC9902	3d (CA)	RC	RC	RC	RC	RC	RC	RC	RC	RC	NC	RC	RC	RC
BL107	3d (CA)	RC	RC	RC	RC	RC	RC	RC	RC	RC	NC	RC	RC	RC
RS9916	3d (CA)	RC	RC	NC	RC	RC	RC	RC	RC	RC	NC	RC	RC	RC
CC9605	3c	RC	RC	RC	RC	RC	RC	RC	RC	RC	RC	RC	RC	RC
WH8102	3c	RC	RC	RC	RC	RC	RC	RC	RC	RC	RC	RC	RC	RC

RC, gene located within the PBS rod gene cluster. In some strains, homologs of these genes are found elsewhere in the genomes (NC). CA, type IV chromatic adapter.

### Phycobilisome linker polypeptides

The core-membrane linker L_CM_, encoded by *apcE*, possesses three predicted repeat (or linker-like) domains in all marine *Synechococcus *except strains CC9311 and RS9916, in which L_CM _has four such domains. RCC307 has the shortest L_CM _sequence (953 amino acids) compared to the other strains due to shorter Arm2 and Arm3 regions (see [15,41] for details on L_CM _domains). Besides the PC-associated linker genes found in the rod gene region of both blue-green strains (Figure 4), WH5701 has a third *cpcC *homolog (*cpcCIII*) located elsewhere in the genome that potentially encodes a chimeric protein since it has an extended carboxyl terminus showing strong similarity to CpcD. None of the PE-containing strains possesses *cpcC *and *cpcD *homologs. In all marine *Synechococcus *genomes, the rod-core linker gene *cpcGII *is found in the PBS rod region whereas *cpcGI *is found outside this cluster. A third *cpcG *gene copy, which we refer to as *cpcGIII*, is present elsewhere in the genomes of BL107, CC9902, CC9311 and CC9605.

The total number of putative PE-associated linker genes varies from zero in the blue-green strains to six in the group constituted by the chromatic adapters and RCC307 (Table 2 and Figure 4). The location of the *mpeE *linker gene appears more variable than the other PEII genes, as it can be found either in the PBS rod gene region (for example, upstream of *cpcGII *in CC9311 or downstream of *cpcGII *in RS9916) or a few genes upstream of this region (in RCC307, BL107 and CC9902) or even in a totally different location of the genome (in CC9605).

Surprisingly, the PEII-lacking strain WH7805 possesses a homolog of *mpeD*, a gene known to encode a chimeric protein made of a PEII-associated linker (amino terminus) and a PEI-associated CpeD-like linker (carboxyl terminus) [19]. However, closer examination of the amino-terminal part of this protein in WH7805 reveals a relatively low similarity with other MpeD sequences and a notable deletion of the region corresponding to amino acids 43-59 in *Synechococcus *sp. WH8102 [19] that is conserved in all other MpeD sequences (Additional data file 2). This region includes two cysteinyl residues involved in linking a PUB chromophore via a Δ2,3 double bond, a type of chromophorylation typical of PEII-associated linker polypeptides [21]. *Synechococcus *sp. WH7803 also lacks the *mpeC *gene, which encodes the distal PEII-associated linker polypeptide in other strains [19,21]. Finally, both chromatic adapters and RCC307 have, outside the PBS core region, an additional gene potentially encoding a PEII-associated linker (Table 3). In phylogenetic trees made with all PEII linkers (Additional data file 3), these sequences are both related to the amino terminus of MpeD but are split between two distinct gene clusters, one gathering BL107, CC9311 and CC9902, which we propose to name MpeF, and the other gathering RS9916 and RCC307, which we propose to name MpeG.

In order to compare further the linker composition of marine *Synechococcus *strains and determine whether they are all present in the PBSs, we performed a lithium dodecyl sulphate (LiDS)-PAGE analysis of intact PBSs. The Coomassie stained gel shown in Figure 5 displays the PBS proteins of two to three strains per pigment type. For WH7803 and RCC307, a Tris-tricine running buffer provided a better separation of the linker polypeptides than Tris-glycine (Figure 5, right). For strains WH5701, WH7805, WH7803, RCC307 and RS9916, all linker polypeptide bands (except ApcC and CpcD, which are not detectable under these electrophoresis conditions) were cut out from the gel and then identified by mass spectrometry (Table 2). In all five strains, the upper band proved to be the core-membrane linker L_CM_, often accompanied by its degradation product L_CM_', making a band of lower apparent molecular weight. As expected, RS9916, which has an extended *apcE *gene sequence, possesses the L_CM _band of lowest electrophoretic mobility. Although the rod-core linker CpcGI was systematically present in all four strains, no CpcGII was detected by mass spectrometry, suggesting either that the *cpcGII *gene is expressed at a much lower level than *cpcGII *or that CpcGII is not present in the PBS fraction of these strains. It is worth noting though that we previously observed CpcGII (co-migrating with CpcGI) in a PBS fraction from *Synechococcus *sp. WH8102 [19]. Interestingly, we identified all three predicted PC rod linkers in WH5701, including the CpcCD-like protein, which is not found in the RS9917 genome. Furthermore, all PEII linkers predicted in WH7803, RCC307 and RS9916 were detected by mass spectrometry, except the products of the *mpeF *gene of RS9916 and of the *mpeG *gene of RCC307 (Table 1). This suggests that either these two potential linker genes are not expressed in our standard culture conditions or their products are undetectable on Coomassie-stained LiDS-PAGE gels due to some inherent biochemical properties.

### Lyases, lyase-isomerases and related genes

Four types of phycobilin lyases, enzymes involved in the chromophorylation of phycobiliproteins, have been characterized so far. One of these, the heterodimeric CpcE/F complex, reversibly ligates a PCB molecule to Cys-84 of the α-subunit of C-PC [42,43]. Two genes with strong homology to the characterized *cpcE *and *cpcF *genes of *Synechococcus *spp. PCC 7942 [44] and PCC 7002 [45] are found near the 3'-end of the rod gene region in 7 out of the 11 marine *Synechococcus *genomes. We have called these *cpcE-F *in the two C-PC-containing strains (RS9917 and WH5701) and *rpcE-F *in WH7803, CC9311 and CC9902, in agreement with the nomenclature proposed by Wilbanks and Glazer [37]. Indeed, *Synechococcus *sp. WH7803 (as well as WH8020 and WH8103) possesses a R-PCII type PC that has a PEB at α-84 [16]. Though we have called these genes *rpcE/F *in strains WH7805 and RCC307 as well (Additional data file 1), it is worth noting that in phylogenetic trees made with concatenated CpeE-F or RpcE-F protein sequences using *Gloeobacter violaceus *as an outgroup, these two strains cluster with RS9917 and WH5701, with only moderate bootstrap support (Additional data file 4). Both CpeE/F and RpcE/F lyases from marine *Synechococcus *possess all sites described by Zhao and coworkers [46] to be important for the activity of CpeE/F in *Fischerella *sp. PCC 7603 (a.k.a. *Mastidocladus laminosus*), so they cannot be differentiated on this basis. In the four other *Synechococcus *genomes, including the high PUB:PEB strains WH8102 and CC9605 and the chromatic adapters BL107 and RS9916, these two lyase genes are replaced by a single fusion gene that we propose to call *rpcG *(Table 3). The amino- and carboxy-terminal parts of the *rpcG *gene product show strong homology to the PecE and PecF of *Fischerella *sp., respectively, the two subunits of a PCB lyase-isomerase, which binds a PCB to Cys84 of the phycoerythrocyanin α-subunit and concomitantly isomerizes it into phycoviolobilin [47,48]. A conserved motif 'NHCQGN' shown to be crucial for the isomerase activity of *Fischerella *PecF is present in the carboxyl terminus of the four marine *Synechococcus *RpcG sequences (for example, positions 361-366 of SYNW2005 in WH8102). This suggests that RpcG is also a phycobilin lyase-isomerase, although several other sites defined as potentially important for the activity of the PecE/F enzyme in *Fischerella *sp. [49] are not conserved in those sequences.

An ortholog of *cpcT*, shown in *Synechococcus *sp. PCC 7002 to encode a lyase catalyzing the binding of PCB at Cys153 of the C-PC β-subunit [50], is found in WH5701, RS9917, WH7805, RCC307 and RS9916 (Table 3). This gene belongs to a family of three paralogs, including *cpeT*, first described in the PE gene cluster of *F. diplosiphon *[39] and located at a similar position in all PE-containing marine *Synechococcus *(Figure 4). An uncharacterized gene located near the 5'-end of the PBS rod gene cluster of all PE-containing strains except RCC307 also belongs to this family. We propose to name this gene *rpcT*, since it is present in the PC-specific gene region of WH7803, which possesses R-PCII. Thus, most marine *Synechococcus *strains possess either *cpcT *or *rpcT*. Surprisingly, the RS9916 strain possesses both genes, confirming their paralogous nature (Additional data file 5).

Marine *Synechococcus *possess another family of three paralogous lyase genes. One of them encodes a lyase that was first characterized in *Nostoc *sp. PCC 7120 as catalyzing the binding of PCB at β-84 of both C-PC and phycoerythrocyanin [51]. More recently, this enzyme was shown to have an even larger spectrum of activity, since it is also able *in vitro *to bind PCB at Cys84 of all AP subunits (that is, ApcA, B, D and F) from *Nostoc *sp. as well as PEB at Cys84 of both α- and β-PE subunits (that is, CpeA and B) from *F. diplosiphon *[52]. Surprisingly, Zhao and co-workers have called this lyase 'CpeS1' though there is no PE in PCC 7120 and its best hit in the marine *Synechococcus *protein databases is not the product of the *cpeS *gene (located immediately upstream of *cpeT *in the PE gene sub-region; Figure 4), but the product of a gene found in tandem with *cpcGI *in all *Synechococcus *strains, including blue-green, PE-lacking strains. So, we suggest to rename it *cpcS *(Table 3, Figure 4 and Additional data file 6). Surprisingly, the *cpcS *gene is split into two different reading frames in WH5701. This is likely a sequencing error, because absence of chromophorylation at Cys84 in all AP and in β-PC subunits would likely render the energy transfer through these phycobiliproteins very poorly efficient. An uncharacterized gene located upstream of the *pebA-B *operon (Figure 4) constitutes the third member of this family of paralogous lyase genes (Additional data file 6), and we propose to name it *cpeU*.

PE-containing *Synechococcus *possess several genes in the PEI or PEII gene sub-regions that encode proteins showing homology to other types of lyases, likely involved in binding phycobilins to one or both PEs. These lyases include CpeY and CpeZ, which in *F. diplosiphon *were presumed to be subunits of a heterodimeric lyase, binding PEB to PE α- or β-subunits [53], but the precise site specificity of this enzyme is hitherto unknown. The *mpeU *and *mpeV *genes, which were first observed in *Synechococcus *sp. WH8020 by Wilbanks and Glazer [37], likely encode two additional lyases. These paralogous genes are both present in the chromatic adapters and in RCC307, whereas WH7803 and WH7805 have only *mpeV *and the high PUB:PEB strains only *mpeU *(Table 3). Finally, we found two novel, paralogous lyase genes, again closely related to one another and more distantly related to *cpeY*. We propose to name these genes *mpeY *and *mpeZ*. Contrary to CpeY and CpeZ, the products of these putative lyase genes likely do not form heterodimers, given their distinct phyletic profiles (Table 3). Indeed, *mpeY *is found in the PEII-specific sub-region of all PEII-containing strains (Figure 4) whereas *mpeZ *is found only in the genomes of the chromatic adapters and of RCC307, outside the PBS gene clusters.

### Conserved hypothetical genes located in the phycobilisome gene region

Table 4 reports the phyletic profile of 13 conserved hypothetical genes associated with the PBS rod region of all (or a majority of) strains. Many of them are seemingly specific to marine *Synechococcus *while some are found in other cyanobacterial genera, including *Prochlorococcus *and/or *Gloeobacter*. It is worth noting though that there are still very few genomes of phycoerythrin-containing cyanobacteria in current databases and it is likely that homologs will be found in those as they become available. In this study, we have given these genes the provisional names *unk1-13*, until a more complete characterization is performed.

As already mentioned, the *unk1 *gene is located upstream of the PBS rod region in all strains except RCC307, in which *unk1 *is located elsewhere in the genome. Another unknown gene (*unk2*) immediately follows *unk1 *in most PE-containing strains (in RCC307, it is the first gene of the PBS rod gene region). The *unk2 *gene is found three genes downstream of *unk1 *in the two blue-green strains. The predicted Unk2 protein sequence generally shows a fairly large variability among the different *Synechococcus *strains, although the BL107 and CC9902 sequences are very closely related (91% identity at the amino acid level). Both Unk1 and Unk2 are short proteins with no recognizable motifs. The *unk3 *gene is associated with the PBS rod region in only four out of the eleven genomes and encodes a protein with six putative transmembrane helices. It is therefore probably not directly related to PBS structure. The *unk4 *gene is present upstream of *aplA *in all PE-containing strains and directly upstream of *cpcGII *in WH7805, which lacks *aplA*. The *unk5 *gene, generally located downstream of *cpcGII*, has the same phyletic profile as *unk4 *(Table 4) and its product possesses pentapeptide repeat motifs. Though very short (57-61 amino acids), the Unk6 protein is very well conserved among the PE-containing *Synechococcus*. A cluster of three consecutive short and conserved hypothetical genes (*unk7-9*) is found only in PEII-containing strains. Localization of these genes in a PEII-specialized sub-region strongly suggests that they are involved in some still unknown function specifically related to PEII. The predicted proteins Unk7 and Unk8 both possess a motif of unknown function (PF07862) also found in the product of a gene located in the *nif *cluster of several cyanobacteria as well as in the nitrogen-fixing proteobacterium *Azotobacter vinelandii *[54]. Surprisingly, in WH7803, *unk7 *and *unk8 *are fused and reversed with regard to *unk9*. This suggests that these genes encode two subunits of the same heterodimeric complex. In the high PUB:PEB strains WH8102 and CC9605, the PEII-specialized region ends with *unk10*, which is strongly conserved between these strains (90% identity at the amino acid level). Homologs of *unk10 *are also found in the genomes of the chromatic adapters and in RCC307 but outside the PBS rod gene region and have only about 49% identity with sequences of the high PUB:PEB strains. Located in the PEI-specific region, the translated *unk11 *gene is very variable in length and sequence (especially the 3'-end) among marine *Synechococcus *strains. In contrast, the neighboring gene *unk12 *displays low sequence variability between strains. Finally, the *unk13 *gene, though strongly conserved, was not correctly modeled in WH8102, in which a wrong open reading frame (ORF; *SYNW2018*) was predicted in a different reading frame. The *unk12 *gene was previously known and was called *orf140 *in WH8020 by Wilbanks and Glazer [37], who sequenced the 3'-end of the PBS rod gene region from *mpeB *to the phosphatase. By remodeling this region, we confirmed that the *unk11 *and *unk13 *genes are also present in this strain and were incorrectly assigned by these authors. Though partial, the organization and gene content of this region in WH8020 [37] is clearly similar to that of chromatic adapters (Figure 4), and this is confirmed by the ability of this strain to chromatically adapt (Table 1; see also [33]).

### Phylogeny of phycobilisome genes

**Figure 6:**
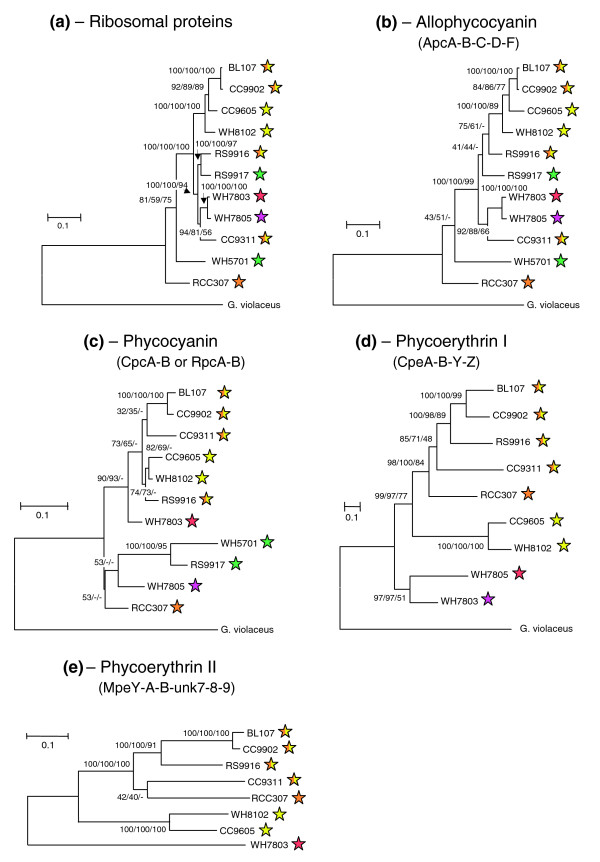
ML trees made with concatenated amino acid sequences of **(a) **all 51 ribosomal proteins (6,754 amino acid positions), **(b) **the AP proteins ApcA-B-C-D-F (710 amino acid positions), **(c) **the PC proteins CpcA-B or RpcA-B (332 amino acid positions), **(d) **the PEI proteins CpeA-B-Y-Z (943 amino acid positions) and **(e) **the PEII proteins MpeA-B-Y and Unk7-8-9 (1,007 amino acid positions). The first four trees are rooted with corresponding proteins from the primitive, freshwater cyanobacterium *Gloeobacter violaceus*, taken as an outgroup. The PEII tree is unrooted since these proteins are specific for marine *Synechococcus *spp. Numbers at internal branches correspond to bootstrap values for 1,000 replicate trees obtained with ML/NJ/MP methods. Colored stars indicate the pigment type of each strain (see Figure 1 for color code).

Both PBS gene complement and organization in the genome are very similar for strains belonging to a given pigment type, independent of their position in phylogenetic trees based, for instance, on the 16S rRNA gene [23,34]. Thus, we wondered whether the phylogeny of PBS genes could differ from the core genome phylogeny. To answer this question, we built phylogenetic trees based on concatenated protein sequences of each phycobiliprotein type and compared them with reference trees made with all concatenated ribosomal proteins, which are good representatives of the core genome (Figure 6). Concatenation generally allows building phylogenies that are more robust when sequences are strongly conserved, as is the case for phycobiliproteins. Still, maximum parsimony (MP) analyses generally provided more variable results than maximum likelihood (ML) and neighbor joining (NJ) analyses due to a relatively low number of informative sites. Whenever possible, we used the primitive, PE-containing, freshwater cyanobacterium *Gloeobacter violaceus *as an outgroup to root our trees, in order to better understand evolution of PBSs within the marine *Synechococcus *group.

The phylogenetic trees obtained with concatenated proteins encoding the AP components (ApcA-B-C-D-F; Figure 6b) share many characteristics with those based on ribosomal proteins (Figure 6a). In both cases, RCC307 and WH5701 are isolated on two long branches well apart from all other strains. Furthermore, WH7803 and WH7805 on the one hand, and CC9902 and BL107 on the other, appear closely related to one another. The only variable positions are those of the closely related strains RS9916 and RS9917, which cluster on the same branch as WH7803, WH7805 and CC9311 in the ribosomal tree, and at the base of the branch bearing BL107, CC9902, CC9605 and WH8102 in the AP tree, but with relatively low bootstrap support in the second case.

The phylogenetic trees of concatenated PC α- and β-subunits (CpcGII was not included because mass spectrometry analyses suggested it may not be part of the PBS; Table 2) show a number of differences relative to the AP tree, including the fact that the two blue-green strains group together (with high bootstrap support) apart from all others (Figure 6c). This is consistent with the fact that they both have C-PC (binding only PCB), whereas all other strains have a PC form binding both PCB and PEB. The relative positions of WH7805 and RCC307 varied between phylogenetic methods. WH7805 is known to contain R-PCIII [17] and this is probably the case for RCC307 as well, based on their similar PC lyase gene content, including *cpcS*, *cpcT *and *rpcE-F *(Table 3). All strains containing R-PCII (or possibly another, unidentified PC form, for those strains possessing *rpcG*; Table 3) formed a well-supported cluster with both ML and NJ methods, though the relative positions of CC9311 and RS9916 were variable within this cluster.

The phylogeny obtained for the concatenated PEI proteins CpeA-B-Y-Z - addition of Unk12 did not significantly alter the tree topologies, but gave lower bootstrap support (data not shown) - fits well with the pigment types, as defined in Table 1. Indeed, the two high PUB:PEB strains group together, well apart from the other PE-containing strains. RCC307 is found at the base of a cluster formed by chromatic adapters (Figure 6d), consistent with the fact that all these strains share a similar PBS gene complement and organization. Likewise, strains WH7803 and WH7805 group together, consistent with the similar organization of their PEI-like region, with *cpeZ *being located downstream of *mpeV *instead of upstream of *cpeY *as in all other PE-containing strains (Figure 4).

Phylogenetic trees obtained with the concatenated PEII proteins MpeA-B-Y and Unk7-9 - inclusion of Unk7-9 does not alter the tree topologies obtained with the sole MpeA-B-Y sequences but provides better bootstrap support - are shown without an outgroup, since this phycobiliprotein form is not found in freshwater cyanobacteria. Still, these trees are globally similar to those obtained with PEI proteins, with three main clusters, one gathering the medium PUB:PEB strain RCC307 and the chromatic adapters, one gathering the two high PUB:PEB strains, whereas the low PUB:PEB strain WH7803 clusters apart from all others.

## Discussion

### Comparative genomics reveal novel genes involved in phycobilisome metabolism

We have identified and compared a number of genes potentially involved in the synthesis and chromophorylation of PBSs in a variety of sequenced marine *Synechococcus *strains spanning all PBS pigment types known so far in this group. Strains displaying different pigment types have different gene complements with a considerable increase in complexity from type 1 (WH5701 and RS9917) to type 3d (chromatic adapters). Synthesis of rods entirely composed of PC, as found in the first type, requires at least 15 genes. This includes two *cpcB-A *operons encoding C-PC α- and β-subunits, two rod-core linker genes (*cpcGI *and *cpcGII*), two *cpcC *and one *cpcD *rod linker genes (in WH5701, an additional *cpcC *gene, *cpcCIII*, was in fact found to be a *cpcC/D *gene chimera), four genes encoding three different lyases (CpcE/F, CpcS and CpcT) and the PCB biosynthesis gene *pcyA*, which encodes the PCB:ferredoxin oxidoreductase [55]. Whether *unk1 *and *unk2*, usually found at or near the 5'-end of the PBS rod gene region (Figure 4), are also involved in PC metabolism awaits experimental checking. An additional *cpcB *gene, absent from other blue-green cyanobacteria such as *Synechococcus *sp. PCC 7942 or *Synechocystis *sp. PCC 6803, is found unlinked to other PBS genes in both WH5701 and RS9917. While the three *cpcB *copies are almost identical in RS9917, the isolated copy is somehow divergent from the other two in WH5701. This may indicate a recent change in function. All PEII-containing strains possess an AP-like gene encoding a protein derived from a phycobiliprotein, the homolog of which, *aplA*, was shown in *F. diplosiphon *to encode a photoreceptor not linked to the PBS [40]. So it is possible that the additional CpcB found in the blue-green strains might have a similar function though, contrary to AplA, this protein appears to have retained the ability to interact with the α-PC subunit. Indeed, amino acids involved in maintaining these interactions [56] are conserved in all CpcB copies.

By comparing the PBS gene complement of strain WH7805 with that of blue-green strains, it appears that the occurrence of a single PEI-like PE type in the rod necessitates at least 19 genes. This includes one set of PE α- and β-subunit encoding genes, three linker genes (*cpeC*, *cpeE *and a *mpeD*-like gene), six putative lyase genes, two genes involved in PEB synthesis (*pebA *and *pebB*) and a number of novel genes of yet unknown function, including *unk5*, *6*, *12*, *13 *and perhaps *unk11*. Indeed, all these *unk *genes are specific to PE-containing *Synechococcus *and all but *unk11 *are well conserved. Despite its tiny size, explaining why it has often been missed by annotation software, *unk6 *is likely a true gene since it is also present in all *Prochlorococcus *strains (data not shown). In both *Prochlorococcus *and marine *Synechococcus *spp., *unk6 *is located upstream of the putative phycobilin lyase gene *cpeS*.

Acquisition of a second PE type, PEII, involves comparatively few additional genes, from six in WH7803, including *unk7/8 *and *unk9*, up to twelve genes in type IV chromatic adapters and RCC307 (*mpeA*, *B*, *C*, *D*, *E*, *F or G*, *U*, *Y*, *Z *and *unk7*, *8*, and *9*), among which the seven underlined genes are novel PEII genes. The fact that PEII synthesis and regulation processes require fewer genes than for PEI implies that several genes involved in these processes are common to both PE forms. This obviously includes the PEB synthesis genes *pebA/B*, but likely also a number of lyase genes.

### Predicting lyase gene function

Examination of the number, phylogenetic relatedness and phyletic profiles of all predicted lyase genes (Table 3) can give us clues about the possible functional specificity of these enzymes. The number of chromophore binding sites on the α- and β-subunits of phycobiliproteins varies from seven in pigment type 1 - that is, four in AP (ApcA, B, D and F subunits have one each) and three in PC (one in CpcA/RpcA, two in CpcB/RpcB) - up to eighteen in PEII-containing strains - that is, four in AP, three in PC, five in PEI (two in CpeA, three in CpeB) and six in PEII (three in MpeA, three in MpeB) -. Furthermore, it is thought that type IV chromatic adapters can have either PUB or PEB at two chromophore binding sites of MpeA [34]. Finally, while the chromophorylation of L_CM _with PCB is thought to be auto-catalyzed and, thus, likely does not require any lyase activity [49], chromophorylation with PUB of the two to four PEII rod linkers (Table 2) probably requires one or several specific PUB lyases (or PEB lyase-isomerases). By comparison, the number of predicted proteins showing homology to known lyases varies from 3 in blue-green strains up to 12-13 in RCC307 and chromatic adapters.

All three phycobilin lyases identified in the genomes of *Synechococcus *spp. WH5701 and RS9917 (Table 3) have characterized homologs in freshwater cyanobacteria. This reduced set of lyases is most likely sufficient to catalyze the chromophorylation with PCB of all AP and C-PC binding sites. Indeed, the CpcS lyase (named 'CpeS1' by Zhao and coworkers [51,52]) is active on almost all α-84 and β-84 cysteinyl residues. The only exception is C-PC α-84, chromophorylation of which is under the control of the heterodimeric lyase CpeE/F [42,43]. Chromophorylation of the last cysteinyl residue, that is, C-PC β-155, is catalyzed by another specific lyase, CpcT [50]. A fairly large difference exists between the sequences and active sites of the CpcE/F lyase, which binds PCB (a type 1 chromophore carrying a Δ3,3^1^-ethylidene group and a single bond between C-2 and C-3) to C-PC α-84, and those of the lyase-isomerase PecE/F, which binds phycobiliviolin (a type 2 chromophore carrying a 3-vinyl group and a Δ2,3-double bond) to the homolog position of α-phycoerythrocyanin [47,48]. Thus, the replacement in four *Synechococcus *strains (BL107, RS9916, CC9605 and WH8102) of *cpeE *and *cpeF *genes by a fusion gene encoding a PecE/F-like protein (that we have called RpcG) is quite significant and it is possible that the PC synthesized by these strains binds a type 2 chromophore at α-84. This interesting hypothesis suggests that a better biochemical characterization of the PC found in these strains is needed. Finally, in all PEII-containing *Synechococcus *strains except RCC307 and RS9916, the *cpcT *gene is absent (Table 3) and seemingly replaced by a gene of the same family of paralogs, located in the PC-specific gene cluster (Figure 4), that we have called *rpcT*. Given the presence of the *rpcT *gene (and absence of *cpcT*) in *Synechococcus *sp. WH7803 in which a PEB is bound at β-153 of R-PCII [16], RpcT is a plausible candidate for catalyzing this specific chromophorylation. Surprisingly, RS9916 possesses both CpcT and RpcT paralogs, suggesting it may either bind PCB or PEB at this site.

Predicting the function of lyase genes potentially involved in bilin attachment to PEI and PEII is much more difficult than for PC, given the larger number of binding sites on these phycobiliproteins. The only lyase gene specific to all PEII-containing strains is *mpeY *(Table 3 and Figure 4). The PEII α-subunit has one chromophore-binding cysteinyl residue that has no homolog in its PEI counterpart, α-75. In WH8103 and white light-grown WH8020, α-75 has been shown to bind a PUB [18]. We hypothesize that MpeY could be a PUB lyase (or a PEB lyase-isomerase) involved in the chromophorylation of PEII α-75 with PUB. However, another specific feature of PEII complexes is that they are held together with two to four PUB-chromophorylated linkers (Table 3) so, alternatively, *mpeY *might encode a lyase involved in the PUB chromophorylation of one (or several) PEII rod linker(s).

The presence of two additional lyase genes in chromatic adapters compared to strains exhibiting either pigment types 3a or 3c (Table 2) suggests that this more complex lyase complement is required for type IV chromatic adaptation. Indeed, this process is thought to consist of the reversible exchange of both PEII α-83 and α-140 chromophores from PEB to PUB [34], and not in the differential expression of several sets of phycobiliprotein genes, like in type III chromatic adaptation (see, for example, [57] for a review). The presence of only one set of genes encoding PEI and PEII α- and β-subunits in all genomes of chromatic adapters supports this hypothesis. Because type IV chromatic adaptation implies the conversion of a PEII-B into a PEII-C under blue light (and conversely under white light; Table 1), it is reasonable to assume that chromatic adapters need two more PUB lyases (or PEB lyase-isomerases) than pigment type 3a strains, which permanently have PEB at PEII α-83 and α-140, and two more PEB lyases than pigment type 3c, which permanently have PUB at these two positions. The phyletic pattern of the *mpeV *gene (Table 3) which, besides its occurrence in chromatic adapters, is also present in WH7803 and WH7805 and absent in the high PUB:PEB strains, suggests it could encode a PEB lyase. Conversely, *mpeU *has the reverse phyletic profile and, thus, could encode a PUB lyase (or PEB lyase-isomerase). The specificity of the putative lyase MpeZ is harder to interpret. Surprisingly, the complex PBS gene set found in chromatic adapters is shared by RCC307, which is the sole strain to have pigment type 3b of all marine *Synechococcus *strains screened so far. Indeed, we have determined that all strains except RCC307 described as having a PUB:PEB of approximately 0.7-0.8 by Fuller *et al*. [23] are actually chromatic adapters. This includes strain RCC61 (data not shown), which belongs to the same phylogenetic clade as RCC307 (that is, clade X) [23]. Therefore, we suggest that RCC307 may have lost the ability to chromatically adapt, perhaps due to a mutation in a domain important for lyase activity or the inactivation or loss of some regulatory gene(s) required for this process.

### Predicted models of PBS structures

**Figure 7:**
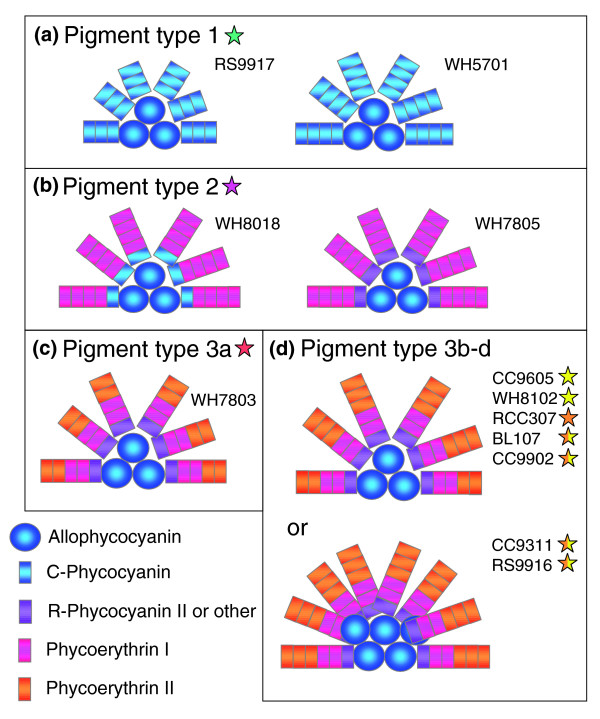
Proposed models of PBS structure for the different *Synechococcus *pigment types and subtypes. PBS cores are generally composed of three cylinders, but in some chromatic adapters possessing an extended L_CM_, it is likely composed of two additional half cylinders (see, for example, [58]). In pigment type 1, rods are composed of C-PC only; in pigment type 2, rods are composed of either C-PC, or R-PCIII and a PEI-like phycobiliprotein; in pigment type 3, rods comprise R-PC and two PE types (PEI and PEII). Cells of the latter pigment type bind PEB and PUB at a low (3a), medium (3b), high (3c) or variable (3d or type IV chromatic adapter) ratio. Colored stars indicate the pigment type of each strain (see Figure 1 for color code).

Most sequenced *Synechococcus *strains have typical PBS cores with three AP cylinders. The presence of an additional L_CM _domain in CC9311 and RS9916 suggests that their PBS core may have two additional half-cylinders, as previously observed in freshwater species such as *Nostoc *sp. PCC 7120 [58]. It is thought that up to eight rods can be bound to such a PBS core (Figure 7). The presence of an extended L_CM _was previously reported from another chromatic adapter, *Synechococcus *sp. M16.17 [34] and one may wonder whether such PBS cores might only occur in this pigment type. An answer to this question awaits screening of *apcE *genes (or of the L_CM _linker size on LiDS-PAGE gels) in a much wider range of strains, as well as direct evidence from electron microscopic images of isolated phycobilisomes.

The large diversity of PBS rod pigmentation observed so far within the marine *Synechococcus *group rests on combinations of at least three PC types (C-PC, R-PCII, R-PCIII), two PEI types (PEI-A/A* and PEI-B) and three PEII types (PEII-A through C) (Table 1). The number and nature of rod linker polypeptides present in the different *Synechococcus *strains can help predict the structure of their PBS rods. Given the striking similarity in pigmentation and gene complement between the freshwater strains *Synechococcus *sp. PCC 7942 or *Synechocystis *sp. PCC 6803 and *Synechococcus *sp. RS9917, the latter strain likely has a very similar PBS rod structure [59,60], that is, three C-PC hexamers (Figure 7a, left). Since *Synechococcus *sp. WH5701 has one more (CpcCD-like) rod linker than RS9917 (Table 2), it is possible that this strain has rods with one additional PC disc (Figure 7a, right).

Like all PE-containing strains, WH7805 lacks the CpcC and CpcD rod linker polypeptides, the absence of which implies it has only a single PC hexamer at the base of each PBS rod. This PC can be of two types depending on strains, C-PC or R-PCIII (Table 1 and Figure 7b). WH7805 has three PE linkers, including a homolog of the long, chimeric rod linker MpeD (Figures 4 and 5) instead of a shorter, CpeD-like linker, like in *F. diplosiphon *[61]. Its amino-terminal moiety is very divergent, however, and does not possess the ability to bind a PUB chromophore, a characteristic common to all PEII rod linkers (Additional data file 2). In the type III chromatic adapter *F. diplosiphon *grown under green light, PBS rods are composed of one PC and three PE hexamers [15,57]. Since they have a MpeD-like rod linker equivalent to two typical rod linkers in length, we suggest that *Synechococcus *pigment type 2 strains might have one more PE disc in their rods than *F. diplosiphon *(Figure 7b).

In a previous paper, we have proposed a model for the structure of PBS rods of the pigment type 3c strain WH8102, which we have predicted to have six hexamers: one PC, two PEI and three PEII [19]. The other high PUB:PEB strain CC9605 appears to have similar PBS rods (Figure 7d). Because it is missing the (distal) linker gene *mpeC*, we assume that the type 3a strain WH7803 has only two PEII hexamers (Figure 7c). Despite the presence of an additional, PEII rod linker gene (*mpeF *or *mpeG*) in chromatic adapters and in RCC307, we found no evidence by mass spectrometry of any such linkers in PBS preparations from RCC307 and RS9916 (Table 2). So, it is very unlikely that these strains have more than three PEII hexamers. Indeed, in this case, they would have a higher whole cell PUB:PEB under blue light than WH8102 or CC9605, whereas this ratio is similar or even lower in chromatic adapters (Table 1). It is possible though that under some specific culture conditions, *mpeF *or *mpeG *could be expressed and that their products could then replace some other PEII linker in the PBS rods.

### New insights into PBS evolution

One major finding from our comparative analyses is that the PBS rod gene complement is highly similar for strains having the same pigmentation (Figure 4 and Tables 2, 3, 4), independent of their position in 16S rRNA [23], 16S-23S rDNA internal transcribed spacer [24] or ribosomal protein phylogenies (Figure 6a), the latter being a proxy for the core genome phylogeny. This is particularly striking for the two blue-green strains RS9917 and WH5701 which, though belonging to different subclusters (5.1 and 5.2, respectively, according to Herdman and co-workers [26]), have a similar gene set and organization of their PBS rod gene region (Figure 4). Phylogenetic trees based on concatenated PC α- and β-subunit sequences from all marine *Synechococcus *also group these two strains together, well apart from all others, in contrast to those obtained with concatenated AP protein sequences, which are globally more consistent with the ribosomal protein phylogeny (Figure 6). Similarly, phylogenies based on PEI and PEII proteins are congruent with the separation of PE-containing strains into pigment types 2 and 3 and subtypes 3a-d, as defined in Table 1. Indeed, they group all chromatic adapters (subtype 3d) together (Figure 6d,e). Furthermore, RCC307 (subtype 3b), which has a similar PBS gene complement and organization, is always found at the base of the chromatic adapter group in PE trees, whereas it appears very distantly related to them in AP trees. Finally, subtypes 3a and 3c strains are found on distinct branches in PE trees and are well separated from chromatic adapters.

Altogether, these data suggest that the different components of the PBS have evolved almost independently from each other in the marine *Synechococcus *group. Indeed, the core of the PBS has seemingly evolved together with the core genome, suggesting that light energy transport from the PBS core to photosystem II is an evolutionarily ancient and conservative mechanism that has not allowed much phenotypic variability during the course of evolution. In contrast, the rod components appear to have evolved through complex episodes of gene duplication, lateral gene transfer and/or gene loss. The latter hypothesis is consistent with recent data from Haverkamp and co-workers [62] showing that phylogenies based on the *cpcB-A *and *cpeB-A *gene sequences notably differ from phylogeny based on 16S rRNA sequences for a variety of *Synechococcus *strains. Acquisition of the first PE (a PEI-like phycobiliprotein) dates back to before the separation of the marine *Synechococcus*/*Prochlorococcus *branch from other cyanobacteria and was likely accomplished by duplication and divergence of ancestral PC genes [63]. In contrast, acquisition of PEII components must have occurred after the differentiation of the marine *Synechococcus *lineage, by duplication and divergence of some PEI genes. Thereafter, transfer of the PEI and/or PEII rod gene cluster might have occurred from one lineage of marine *Synechococcus *to another, possibly by lateral transfer via natural transformation or viruses. The occurrence of photosynthetic genes is frequent in cyanophages [64] and this may include PBS genes, such as the putative lyase *cpeT *gene found in the S-PM2 and Syn9 genomes [65,66] or the *pebA *and *pcyA *genes found in the P-SSM2 and P-SSM4 genomes [67,68]. So far, only individual photosynthetic genes have been found in such phage genomes, not gene clusters. However, it is quite possible that, in some rare cases, much larger genome chunks (for example, covering the whole PEII sub-region) could be conveyed by cyanophages between *Synechococcus *spp. cells belonging to distinct lineages.

## Conclusion

The dazzling colors of marine *Synechococcus *rely on the combination of a few phycobiliprotein forms, which can be assembled into a variety of PBS structures (Figure 7). The variable part of these photosynthetic antennae (that is, PBS rods) is encoded and regulated in large part by a specialized genomic region, which includes a number of genes of unknown function, but rapid progress in elucidating these functions is envisaged using a combination of genetic and biochemical approaches. During the course of evolution, marine *Synechococcus *appear to have acquired more and more sophisticated light-harvesting complexes, from simple C-PC rods to elaborate rod structures comprising three distinct phycobiliprotein types. As a further sophistication, some marine *Synechococcus *strains are able to modulate their PBS absorption capacity to harvest efficiently a larger range of visible light quality. In the present study, we show that these type IV chromatic adapters are much more frequent in culture collections than previously thought, and this might be the case in nature as well, since the distribution of this pigment type in the field is currently unknown. The large diversity of PBS pigmentation found among marine *Synechococcus*, as well as the likely occurrence, during evolution, of PBS gene exchanges between lineages ensuring that this diversity is maintained to some extent at the level of individual lineages, have allowed members of this genus to thrive in almost every possible illuminated marine environment. This may be one of the key reasons explaining the ecological success of the *Synechococcus *group in the marine environment.

## Materials and methods

### *Synechococcus *strains and culture conditions

For biochemical analyses, marine *Synechococcus *spp. strains Almo3, BL107, CC9605, Oli31, RCC307, RS9912, RS9916, RS9917, WH5701, WH7803, WH7805, WH8018 and WH8102 were grown in 8 l polycarbonate flasks (Nalgene, Rochester, NY, USA) in PCR-S11 medium [69] supplemented with 5 mM NaNO_3_. Cultures were grown at 22°C under approximately 15 μmol photons m^-2 ^s^-1 ^white light (Sylvania daylight fluorescent bulbs). To determine their PUB:PEB ratio and their eventual ability to perform type IV chromatic adaptation [34], two to four duplicate 10 ml cultures of a number of strains possessing two PEs were grown in parallel under 15 μmol photons m^-2 ^s^-1 ^white or blue light, prior to spectrofluorometric analyses made during the exponential growth phase. Blue light was obtained by wrapping tube racks with blue filter sheet (filter no. 183 'moonlight', Lee Filters, Andover, England). The origin of strains has been described previously [23] except for RCC307 and BL107, which were respectively isolated from the Mediterranean Sea in June 1999 at 6° 10'E, 39° 10'N at a depth of 15 m by F Partensky and in September 2000 at 13° 33'E, 41° 43'N at the very deep depth of 1,800 m by Laure Guillou (Roscoff, France). All these strains are available from the Roscoff Culture Collection (RCC), Roscoff, France.

### *In vivo *spectrometry

Room temperature excitation fluorescence spectra (with emission at 580 nm) of whole *Synechococcus *cells grown under standard light conditions were recorded with a spectrofluorimeter LS-50B (Perkin Elmer, Waltham, MA, USA), as previously described [32], in order to measure the PUB to PEB fluorescence excitation ratio. *In vivo *absorbance spectra of whole cells were also recorded from 400 to 750 nm with a double monochromator spectrophotometer (Hitachi U-3310) equipped with a head-on photomultiplier detector. Spectra were recorded with a 1 nm interval and 5 nm slit width and normalized at 439 nm (blue absorption peak).

### Phycobiliprotein purification and characterization

PE purification was carried out as described previously [19]. Briefly, after cell breakage in a cooled French press system, a soluble extract devoid of chlorophyll *a *was obtained by differential ultracentrifugation in a buffer containing 10 mM phosphate pH 7.2 and the protease inhibitors EDTA, phenylmethylsulfonyl fluoride, aminocaproic acid and benzamidine, each at 1 mM final concentration. The soluble protein extract was then loaded onto a 0-30% sucrose density gradient and run overnight at 130,000 × *g *at 12°C. Phycobiliproteins were separated from the different colored sucrose gradient fractions on 7% acrylamide isoelectric focusing gels containing ampholyte carriers pH 4-6.5 (Amersham Biosciences, Buckinghamshire, UK). PE bands were cut out of the gel and crushed with an electric grinder in 10 mM tricine buffer pH 7.8. Acrylamide remnants were eliminated by centrifugation. When necessary, samples were concentrated using 30 kDa cut-off membranes (Centricon, Millipore, Billerica, MA, USA). Absorption and fluorescence emission spectra were recorded and corrected as described earlier [19].

### Intact phycobilisome extraction

PBSs were isolated on discontinuous sucrose density gradients in 0.75 M phosphate buffer containing protease inhibitors by the classic sucrose density gradient method [19]. Colored bands were precipitated with 10% (v/v) trichloroacetic acid and resuspended in 3% (w/v) LiDS denaturation buffer. Electrophoresis was carried out overnight using a 10-20% continuous gradient LiDS-PAGE at low amperage (10 mA). After migration, the gel was immersed in 20 mM zinc acetate in order to enhance phycobiliprotein fluorescence, washed with water and visualized under UV light, then stained with Coomassie blue G250. For some selected strains for which genome sequence was available, bands of linker polypeptides were cut out of the gel and identified by mass spectrometry, using the facilities of the 'Unité de Recherche Biochimie et Structure des Protéines', Jouy en Josas, France. Briefly, each gel sample was digested overnight at 37°C in 25 μl trypsin (at 8 μg ml^-1^). Mass spectra were acquired with a MALDI-TOF (Applied Biosystems model Voyager DE super STR, Foster City, CA, USA) equipped with a nitrogen laser with an emission wavelength of 337 nm and run in reflectron mode with an extraction delay of 130 ns. The matrix used was α-cyano-4-hydroxycinnamic acid at 4 mg ml^-1^. Internal calibration was performed with trypsin peptides (842.5090 and 2,211.1040 Da).

### Comparative genomics

Eleven genomes of marine *Synechococcus *spp. were used for this study: The WH8102 (NC_005070), CC9902 (NC_007513) and CC9605 (NC_007516) genomes have been sequenced by the Joint Genome Institute, the CC9311 (NC_008319) genome by The Institute for Genome Research (TIGR), the WH7803 (NC_009481) and RCC307 (NC_009482) genomes by Genoscope (Evry, France) at the request of a consortium of European scientists coordinated by F Partensky, the RS9916 (NZ_AAUA00000000), RS9917 (NZ_AANP00000000), BL107 (NZ_AATZ00000000), WH7805 (NZ_AAOK00000000) and WH5701 (NZ_AANO00000000) genomes by the J Craig Venter Institute in the framework of the Gordon and Betty Moore Foundation Marine Microbial Genome Sequencing Project at the request of an international consortium coordinated by DJ Scanlan.

Gene families from the 11 marine *Synechococcus *were delineated using BLAST [70] with an e-value of 10^-12 ^and the TribeMCL algorithm [71]. Families of orthologous genes either located in the PBS region and/or involved in PBS biosynthesis or regulation were extracted and manually annotated. Non-modeled genes, missed by ORF finding software, were added to the dataset. The corresponding protein sequences were aligned using ClustalW [72] with default parameters and their amino terminus was corrected (that is, extended or shortened) if needed.

### Phylogenetic analyses

Phylogenetic analyses were performed using a variable number of concatenated protein sequences depending on each phycobiliprotein type (see results), allowing the use of longer sequences to reduce the variance in the distance estimates [73]. These sequences were automatically aligned using ClustalW [72]. Alignments were then manually refined and all gaps and highly variable regions (if any) were removed. Phylogenetic trees were generated using three different reconstruction methods: NJ (with PHYLO_WIN [74]), ML (with PHYML v2.4.4 [75]) and MP (with PHYLO_WIN). ML analyses were performed using the Jones Taylor Thornton model and the variability of substitution rates across sites and invariables sites was estimated. Bootstrap values (1,000 replicates) were calculated for all three methods in order to estimate the relative confidence in monophyletic groups and they were all reported on the ML tree used as a reference. Phylogenetic trees were edited using the MEGA4 software [76].

## Abbreviations

AP, allophycocyanin; L_C_, core linker; L_CM_, core-membrane linker; LiDS, lithium dodecyl sulphate; L_R_, rod linker; L_RC_, rod-core linker; ML, maximum likelihood; MP, maximum parsimony; NJ, neighbor joining; ORF, open reading frame; PBS, phycobilisome; PC, phycocyanin; PCB, phycocyanobilin; PE, phycoerythrin; PEB, phycoerythrobilin; PUB, phycourobilin.

## Authors' contributions

CS and FP conceived the study and wrote most of the paper. DJS and FP together coordinated sequencing and annotation of 7 out of the 11 *Synechococcus *genomes used in this study. FP did most comparative genomics analyses and drew genomic regions and phycobilisome models. AD performed the clustering of orthologous genes and set up a web site for annotation for all phycobilisome genes. He also performed the phylogenetic analysis of ribosomal proteins. LG did most other phylogenetic analyses and helped in writing the corresponding part of the manuscript. CS did phycobilisome extractions from selected *Synechococcus *strains, isoelectric focusing gels for purifying intact phycobiliproteins and performed spectrometric analyses. JCT performed LiDS-PAGE analyses of linker polypeptides, cut selected bands out of these gels and supervised mass spectrometry analyses. MO improved the quality of the genome sequence of several *Synechococcus *strains and performed spectrophotometric analyses on intact cells. NB participated in the annotation of phycobilisome genes and checked occurrence of some of them in several unsequenced *Synechococcus*. DJS improved the overall quality of the manuscript.

## Additional data files

The following additional data files are available with the online version of this paper. Additional data file 1 is a table listing genes involved in PBS metabolism or regulation in the 11 genomes of marine *Synechococcus*. Additional data file 2 is an amino acid alignment of the amino terminus of the MpeD linker polypeptide from all sequenced, PE-containing, marine *Synechococcus *spp. Additional data file 3 is an unrooted ML tree based on amino acid sequences of MpeD (amino terminus only) and all other PEII-associated rod linker polypeptides (216 amino acid positions). Additional data file 4 is a ML tree based on concatenated amino acid sequences of CpcE and CpcF homologs in all sequenced marine *Synechococcus *spp. (444 amino acid positions). Additional data file 5 is an unrooted ML tree of the CpcT-CpeT-RpcT protein family (183 amino acid positions). Additional data file 6 is an unrooted ML tree of the CpcS-CpeS-CpeU protein family (163 amino acid positions).

## Supplementary Material

Additional data file 1 Genes involved in PBS metabolism or regulation in the 11 genomes of marine *Synechococcus*. Click here for file

Additional data file 2 Identical residues are shown in yellow type on purple squares, blue type on dark grey indicates that the percentage of conserved residues is >80%, and black type on light grey indicates that the percentage of conserved residues is >60%. Note that PEII-containing strains possess a conserved region containing two cysteinyl residues (highlighted by red boxes), whereas in the PEII-lacking WH7805 strain, this region is missing. This region is involved in the binding of a PUB molecule via a thioether bond linking C-3^1 ^and C-18^1 ^of the chromophore to the two cysteinyl residues. Click here for file

Additional data file 3 Note that the novel, putative linkers found in the chromatic adapters and in RCC307 (Table 3) make two distinct clusters that we have called MpeF and MpeG. Colored stars indicate the pigment type of each strain (Figure 1) and numbers at internal branches correspond to bootstrap values for 1,000 replicate trees obtained with ML/NJ/MP methods, respectively. Click here for file

Additional data file 4 The primitive, freshwater cyanobacterium *Gloeobacter violaceus *PCC 7421 is used as an outgroup. Colored stars indicate the pigment type of each strain (Figure 1) and numbers at internal branches correspond to bootstrap values for 1,000 replicate trees obtained with ML/NJ/MP methods, respectively. Click here for file

Additional data file 5 Colored stars indicate the pigment type of each strain (Figure 1) and numbers at internal branches correspond to bootstrap values for 1,000 replicate trees obtained with ML/NJ/MP methods, respectively. Click here for file

Additional data file 6 Colored stars indicate the pigment type of each strain (Figure 1) and numbers at internal branches correspond to bootstrap values for 1,000 replicate trees obtained with ML/NJ/MP methods, respectively. Click here for file
