# Supplementary figures and images for: Diversity and evolution of phycobilisomes in marine Synechococcus spp.: a comparative genomics study
Source: Genome Biol. 2007 Dec 5;8(12):R259. doi: 10.1186/gb-2007-8-12-r259 (PMC2246261; doi:10.1186/gb-2007-8-12-r259)

## Slide 1
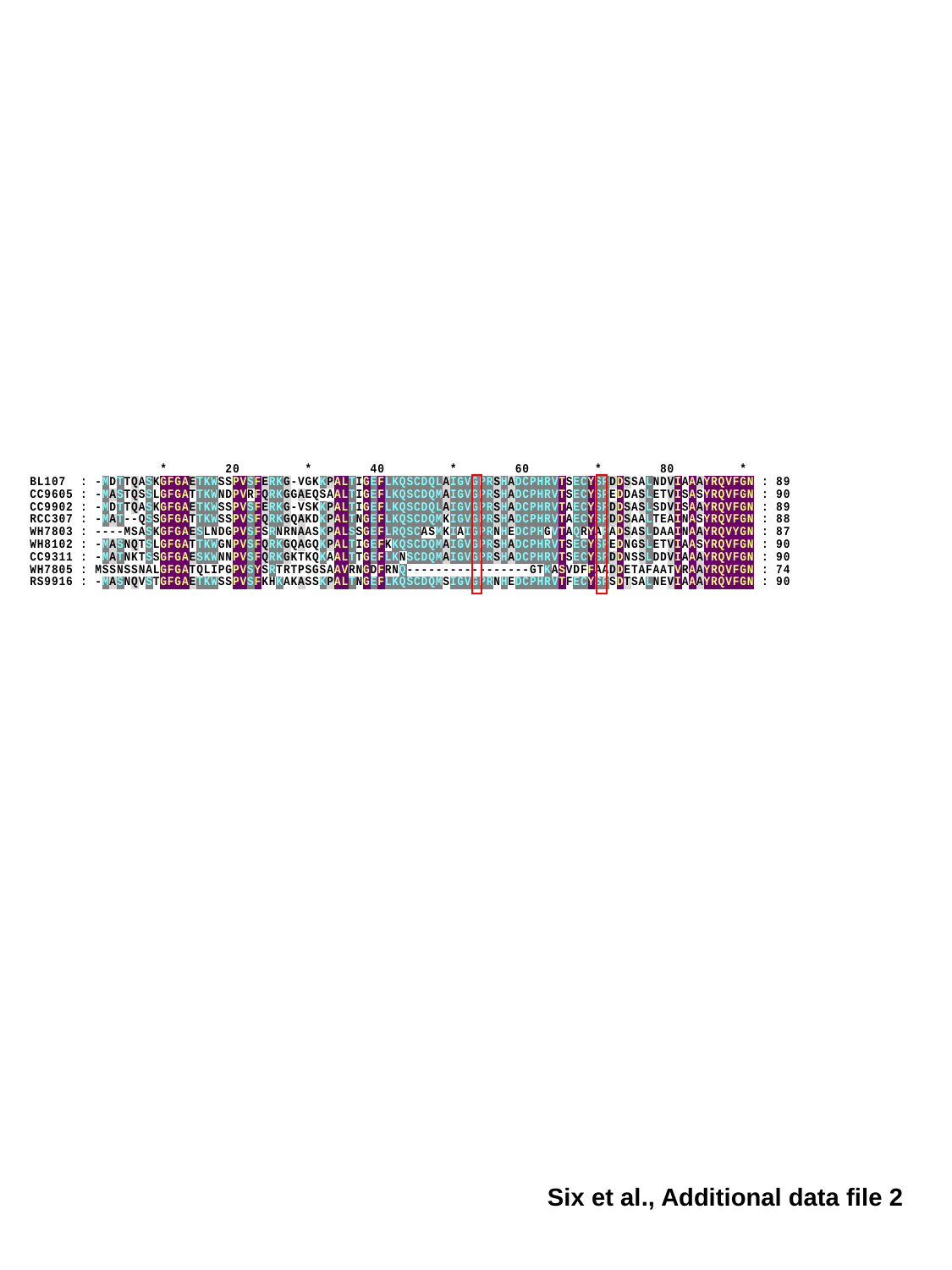

Six et al., Additional data file 2

Supplement: Additional data file 2 — Identical residues are shown in yellow type on purple squares, blue type on dark grey indicates that the percentage of conserved residues is >80%, and black type on light grey indicates that the percentage of conserved residues is >60%. Note that PEII-containing strains possess a conserved region containing two cysteinyl residues (highlighted by red boxes), whereas in the PEII-lacking WH7805 strain, this region is missing. This region is involved in the binding of a PUB molecule via a thioether bond linking C-31 and C-181 of the chromophore to the two cysteinyl residues. [file gb-2007-8-12-r259-S2.ppt]

## Slide 1
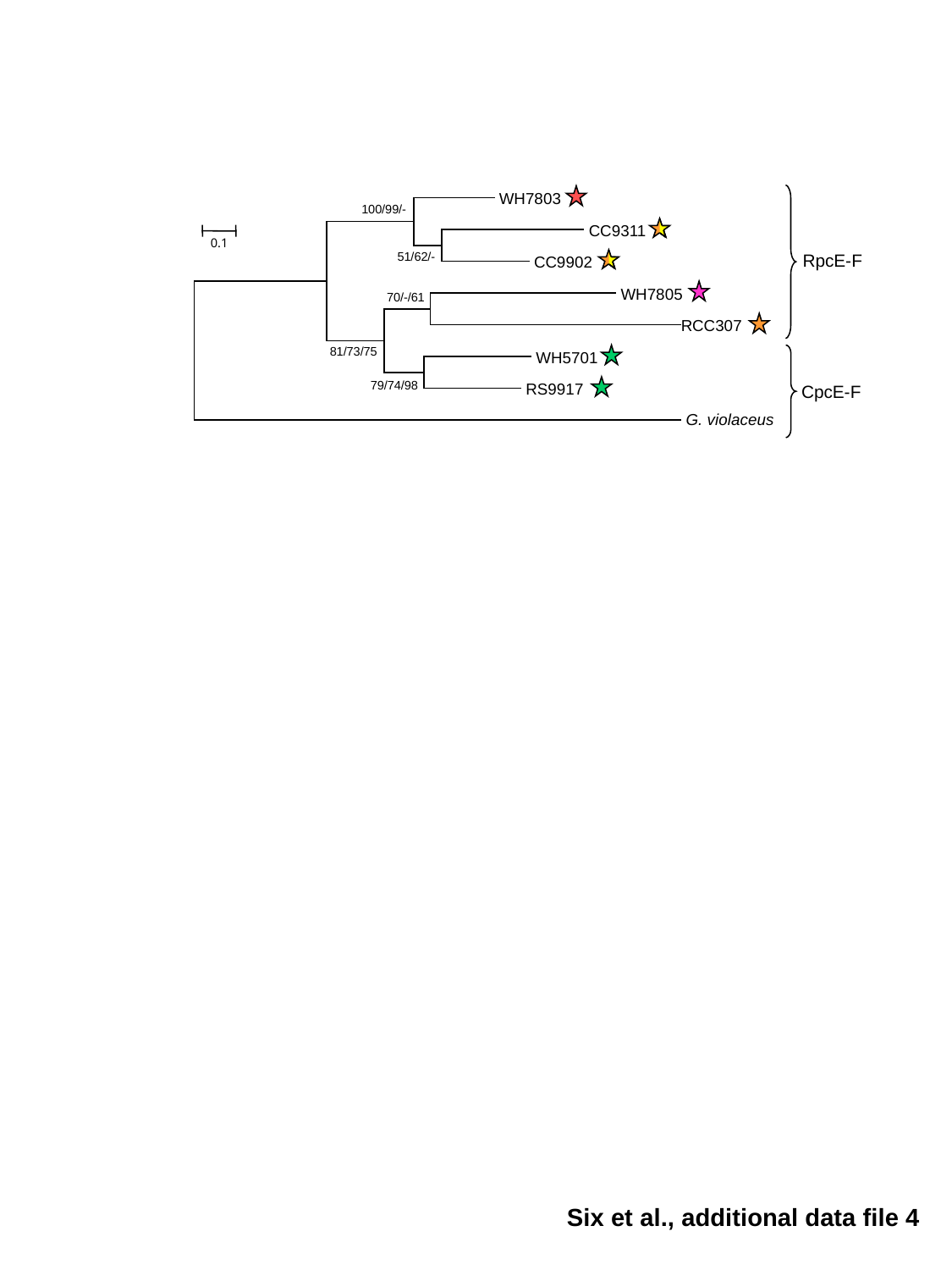

WH7803
100/99/-
 CC9311
0.1
RpcE-F
51/62/-
 CC9902
 WH7805
70/-/61
RCC307
81/73/75
 WH5701
CpcE-F
79/74/98
 RS9917
 G. violaceus
Six et al., additional data file 4

Supplement: Additional data file 4 — The primitive, freshwater cyanobacterium Gloeobacter violaceus PCC 7421 is used as an outgroup. Colored stars indicate the pigment type of each strain (Figure 1) and numbers at internal branches correspond to bootstrap values for 1,000 replicate trees obtained with ML/NJ/MP methods, respectively. [file gb-2007-8-12-r259-S4.ppt]
