# Supplementary material for: Diversity and evolution of phycobilisomes in marine Synechococcus spp.: a comparative genomics study
Source: Genome Biol. 2007 Dec 5;8(12):R259. doi: 10.1186/gb-2007-8-12-r259 (PMC2246261; doi:10.1186/gb-2007-8-12-r259)
Supplement: Additional data file 3 — Note that the novel, putative linkers found in the chromatic adapters and in RCC307 (Table 3) make two distinct clusters that we have called MpeF and MpeG. Colored stars indicate the pigment type of each strain (Figure 1) and numbers at internal branches correspond to bootstrap values for 1,000 replicate trees obtained with ML/NJ/MP methods, respectively. [file gb-2007-8-12-r259-S3.ppt]

## Slide 1
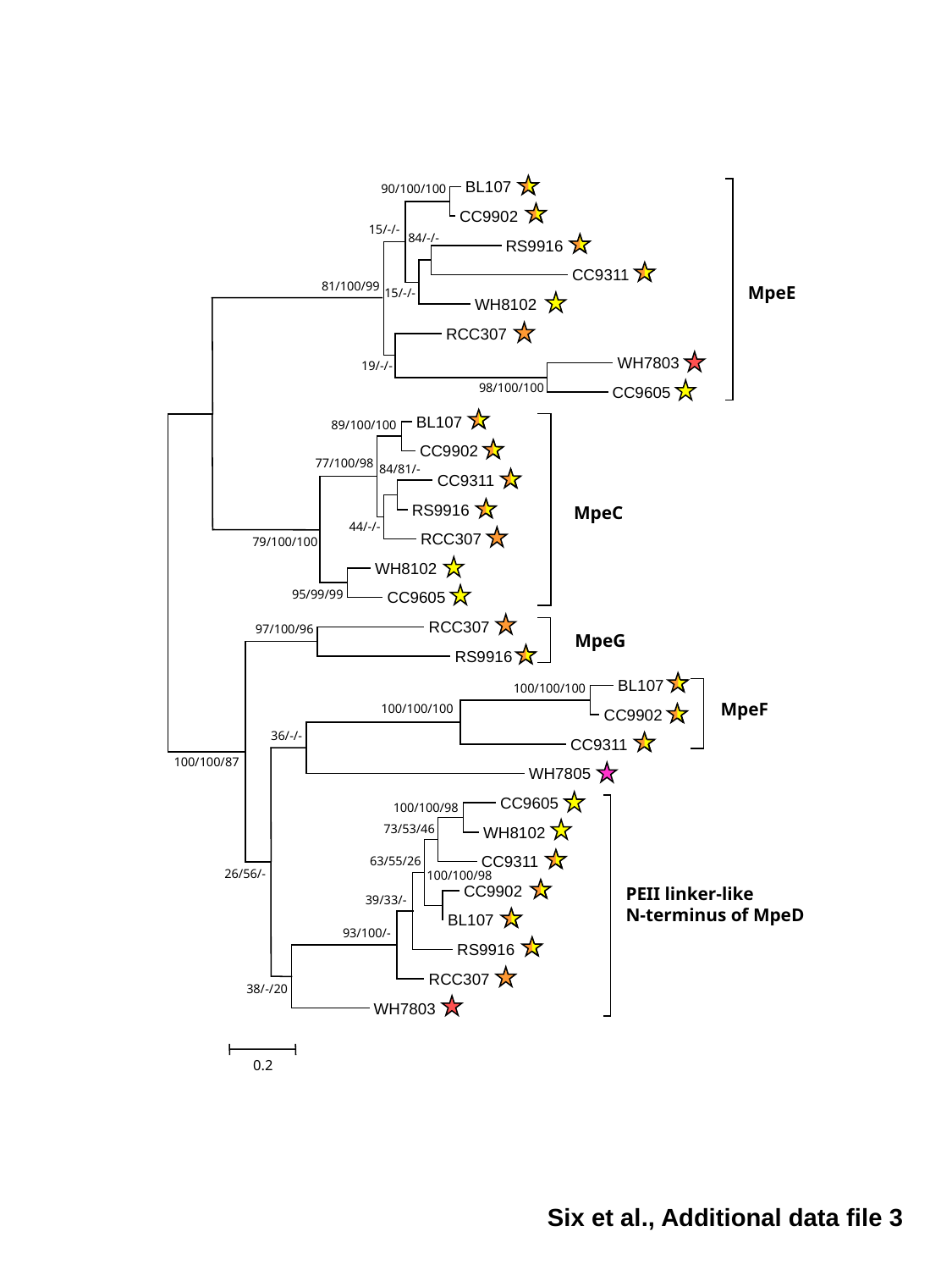

BL107
90/100/100
 CC9902
15/-/-
84/-/-
 RS9916
 CC9311
81/100/99
MpeE
15/-/-
 WH8102
 RCC307
 WH7803
19/-/-
98/100/100
 CC9605
 BL107
89/100/100
 CC9902
77/100/98
84/81/-
 CC9311
 RS9916
MpeC
44/-/-
 RCC307
79/100/100
 WH8102
95/99/99
 CC9605
 RCC307
97/100/96
MpeG
 RS9916
 BL107
100/100/100
MpeF
100/100/100
 CC9902
36/-/-
 CC9311
100/100/87
 WH7805
 CC9605
100/100/98
73/53/46
 WH8102
 CC9311
63/55/26
26/56/-
100/100/98
 CC9902
PEII linker-like
N-terminus of MpeD
39/33/-
 BL107
93/100/-
 RS9916
 RCC307
38/-/20
 WH7803
0.2
Six et al., Additional data file 3
