# Supplementary material for: Diversity and evolution of phycobilisomes in marine Synechococcus spp.: a comparative genomics study
Source: Genome Biol. 2007 Dec 5;8(12):R259. doi: 10.1186/gb-2007-8-12-r259 (PMC2246261; doi:10.1186/gb-2007-8-12-r259)
Supplement: Additional data file 5 — Colored stars indicate the pigment type of each strain (Figure 1) and numbers at internal branches correspond to bootstrap values for 1,000 replicate trees obtained with ML/NJ/MP methods, respectively. [file gb-2007-8-12-r259-S5.ppt]

## Slide 1
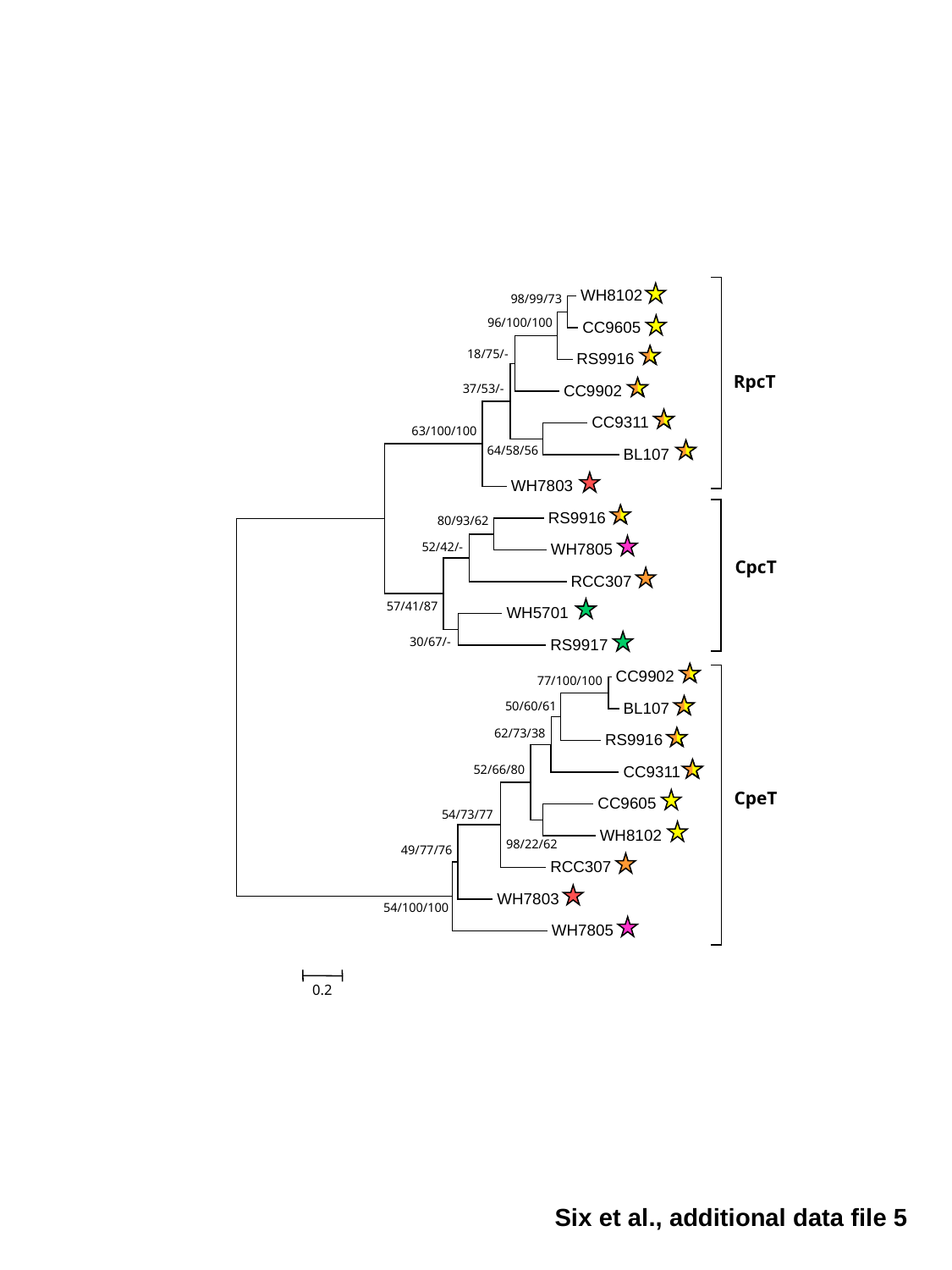

WH8102
98/99/73
96/100/100
 CC9605
18/75/-
 RS9916
RpcT
 CC9902
37/53/-
 CC9311
63/100/100
64/58/56
 BL107
 WH7803
 RS9916
80/93/62
52/42/-
 WH7805
CpcT
 RCC307
57/41/87
 WH5701
30/67/-
 RS9917
 CC9902
77/100/100
 BL107
50/60/61
62/73/38
 RS9916
 CC9311
52/66/80
CpeT
 CC9605
54/73/77
 WH8102
98/22/62
49/77/76
 RCC307
 WH7803
54/100/100
 WH7805
0.2
Six et al., additional data file 5
