# Supplementary material for: Diversity and evolution of phycobilisomes in marine Synechococcus spp.: a comparative genomics study
Source: Genome Biol. 2007 Dec 5;8(12):R259. doi: 10.1186/gb-2007-8-12-r259 (PMC2246261; doi:10.1186/gb-2007-8-12-r259)
Supplement: Additional data file 6 — Colored stars indicate the pigment type of each strain (Figure 1) and numbers at internal branches correspond to bootstrap values for 1,000 replicate trees obtained with ML/NJ/MP methods, respectively. [file gb-2007-8-12-r259-S6.ppt]

## Slide 1
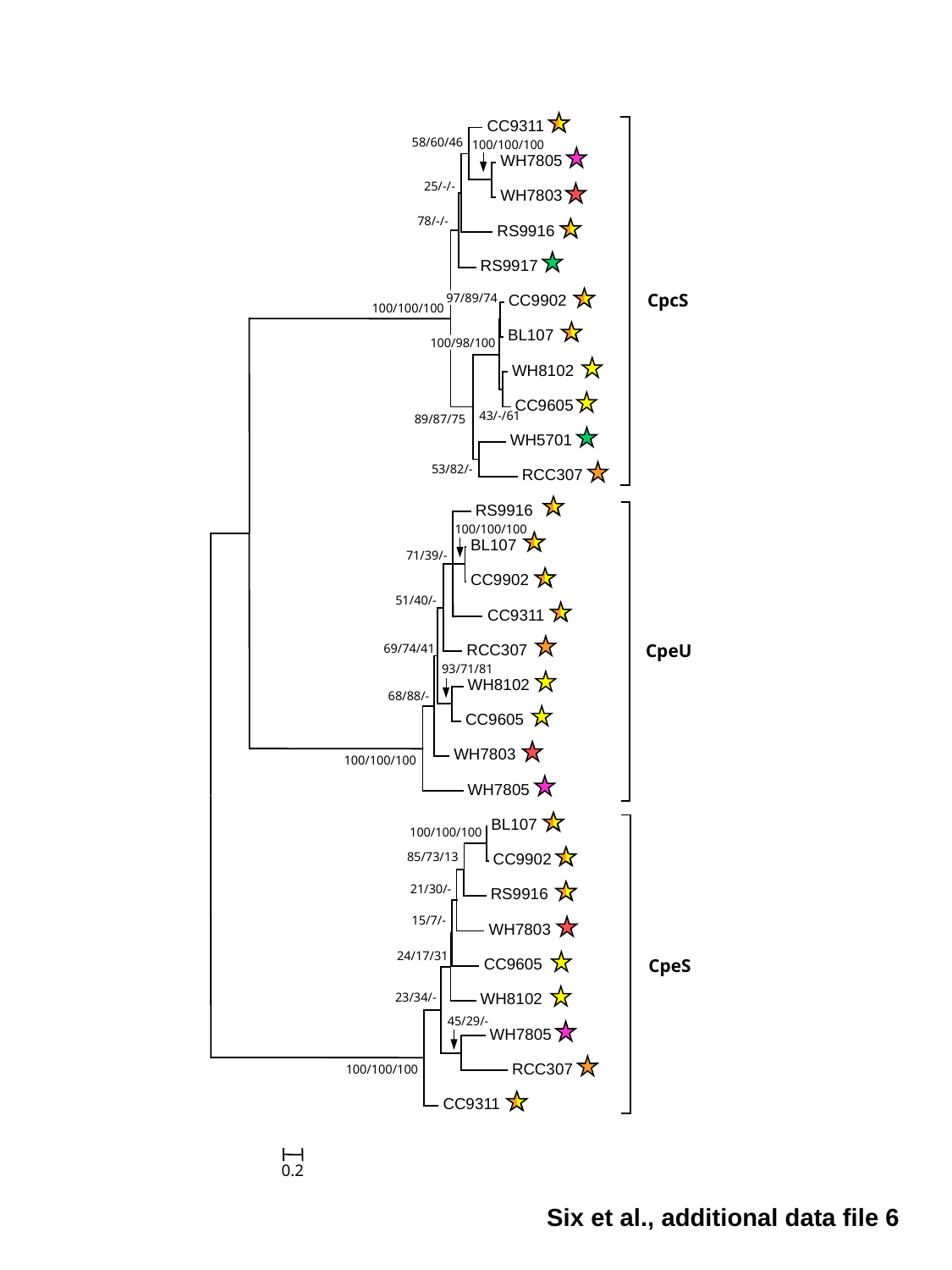

CC9311
58/60/46
100/100/100
 WH7805
25/-/-
 WH7803
78/-/-
 RS9916
 RS9917
CpcS
97/89/74
 CC9902
100/100/100
 BL107
100/98/100
 WH8102
 CC9605
43/-/61
89/87/75
 WH5701
53/82/-
 RCC307
 RS9916
100/100/100
 BL107
71/39/-
 CC9902
51/40/-
 CC9311
 RCC307
CpeU
69/74/41
93/71/81
 WH8102
68/88/-
 CC9605
 WH7803
100/100/100
 WH7805
 BL107
100/100/100
85/73/13
 CC9902
21/30/-
 RS9916
15/7/-
 WH7803
24/17/31
 CC9605
CpeS
 WH8102
23/34/-
45/29/-
 WH7805
 RCC307
100/100/100
 CC9311
0.2
Six et al., additional data file 6
